# Glial and Vascular Plasma Biomarkers Across the Alzheimer's Disease Continuum: An ADNI‐Based Longitudinal Study

**DOI:** 10.1096/fba.2026-00194

**Published:** 2026-09-19

**Authors:** Khodakaram Jahanbin, Alzheimer's Disease Neuroimaging Initiative

**Affiliations:** ^1^ Department of Immunology, School of Medicine Ahvaz Jundishapur University of Medical Sciences Ahvaz Iran

**Keywords:** Alzheimer's disease, biomarkers, blood–brain barrier, glial fibrillary acidic protein, vascular cell adhesion molecule‐1

## Abstract

Alzheimer's disease (AD) is increasingly recognized as a multicellular disorder involving neurovascular unit dysfunction. Investigating glial and vascular biomarkers together may provide a more integrated view of AD biology. This study characterized baseline distributions, interrelationships, and longitudinal trajectories of plasma glial (GFAP, sTREM2) and vascular/endothelial (VEGF, sICAM‐1, sVCAM‐1) biomarkers across the AD continuum. Using Alzheimer's Disease Neuroimaging Initiative (ADNI) data, this retrospective longitudinal cohort study included a covariate‐complete clinical cohort of 2650 participants classified as cognitively unimpaired (CU), mild cognitive impairment (MCI), or AD dementia. Biomarker‐specific analytic samples were determined by assay availability and complete‐case requirements. Associations were evaluated using covariate‐adjusted linear regression and linear mixed‐effects models with participant random intercepts, adjusted for baseline age, sex, education, and APOE ε4 status. Vascular models were additionally refitted with body mass index, systolic blood pressure, antihypertensive and antidiabetic medication use, smoking history, and estimated glomerular filtration rate. Baseline GFAP showed a robust stepwise elevation from CU to MCI to AD (56.3% higher in AD than CU, *q* = 1.7 × 10^−14^; area under the curve 0.82), whereas sTREM2 distributions overlapped across groups. VEGF and sVCAM‐1 were higher in AD than CU (14.1%, *q* = 0.014; 14.7%, *q* = 0.002), with areas under the curve of 0.61 and 0.63 and more than 80% distribution overlap. GFAP increased by 4.50% per year in CU participants and sTREM2 by 2.99% per year, with no significant diagnosis‐by‐time interactions for either. Over a 12‐month interval, sICAM‐1 declined in CU participants, and this decline was attenuated in MCI and AD, while sVCAM‐1 increased in CU participants and showed negative diagnosis‐by‐time interactions. The vascular longitudinal findings were unchanged by adjustment for cardiometabolic and renal covariates, by time‐varying diagnosis, and by separating stable MCI from MCI‐to‐AD converters. Correlations among vascular markers were consistent and well estimated, whereas glial‐vascular correlations were based on 64 to 94 overlapping participants and were not significant. Glial and vascular plasma biomarkers did not progress synchronously across the AD continuum. GFAP showed the strongest and most discriminating diagnosis‐associated elevation. Vascular markers showed statistically robust but small group‐level differences with no individual‐level discriminative utility, and their divergent 12‐month trajectories require replication over longer follow‐up before they can be interpreted as stage‐dependent regulation.

## Graphical abstract

Plasma biomarkers of astroglial activation (GFAP), microglial signaling (sTREM2), and endothelial dysfunction (VEGF, sICAM‐1, sVCAM‐1) are released into the circulation from the neurovascular unit and sampled across the Alzheimer's disease continuum. The inset plots show the contrasting behavior of the two glial markers: GFAP increases stepwise from cognitively unimpaired participants to mild cognitive impairment to Alzheimer's dementia, whereas sTREM2 levels overlap between diagnostic groups.

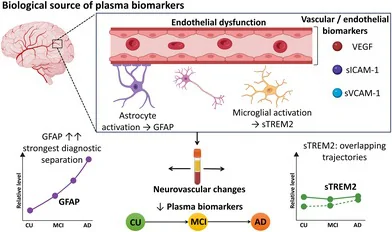

## 1 Introduction

Alzheimer's disease (AD) is biologically defined by amyloid‐β deposition and tau pathology, but it is increasingly recognized as a complex, multicellular disorder that extends beyond these core proteinopathies [1, 2]. Contemporary models emphasize that chronic neuroinflammation, blood–brain barrier (BBB) dysfunction, endothelial injury, impaired perfusion, and neurovascular unit failure evolve alongside amyloid and tau pathology and may contribute directly to neurodegeneration and cognitive decline [3, 4]. This shift has encouraged increasing interest in non‐core AD biomarkers that capture glial activation and vascular dysfunction across the disease continuum. The neurovascular unit provides a useful biological framework for integrating these processes. Therefore, studying glial and vascular biomarkers together may provide a more complete view of AD biology than evaluating each pathway in isolation.

Blood‐based biomarkers are particularly well suited for investigating this multidomain biology. Compared with cerebrospinal fluid (CSF) and positron emission tomography biomarkers, plasma markers are less invasive, more scalable, easier to collect repeatedly, and more practical for large longitudinal cohorts and clinical trials [5, 6].

Among glial markers, glial fibrillary acidic protein (GFAP) has emerged as one of the most promising plasma biomarkers in AD. Plasma GFAP has been associated with amyloid positivity and amyloid burden, even among cognitively unimpaired individuals, suggesting that astroglial activation occurs before substantial clinical impairment [7, 8]. Longitudinal evidence also indicates that elevated plasma GFAP can precede AD‐related cognitive decline and relate to downstream neuropathological plaque and tangle burden [9, 10].

Microglial dysfunction represents a complementary component of AD pathophysiology. Triggering receptor expressed on myeloid cells 2 (TREM2) is an innate immune receptor primarily expressed by microglia and is involved in microglial survival, migration, proliferation, phagocytosis, lipid metabolism, and inflammatory regulation [11, 12]. Soluble TREM2 (sTREM2), released from the membrane‐bound receptor and measurable in plasma and CSF, may therefore reflect microglial activation or immune signaling related to AD pathology [13, 14]. Nevertheless, plasma sTREM2 remains less established than plasma GFAP. Its associations with disease stage, cognitive decline, CSF sTREM2 levels, and other AD biomarkers are still incompletely resolved and occasionally contradictory, highlighting the need for further longitudinal analyses [15, 16].

Vascular and endothelial dysfunction are also increasingly recognized as meaningful contributors to AD rather than incidental comorbid processes. Blood–brain barrier disruption, endothelial activation, impaired cerebral perfusion, vascular inflammation, and leukocyte trafficking may worsen neuronal injury and accelerate cognitive decline [17, 18]. Vascular endothelial growth factor (VEGF) is an angiogenic and hypoxia‐responsive molecule involved in vascular remodeling and endothelial adaptation. In AD, however, VEGF findings have been mixed, with studies reporting lower, higher, or unchanged circulating levels [19, 20].

Soluble intercellular adhesion molecule‐1 (sICAM‐1) and soluble vascular cell adhesion molecule‐1 (sVCAM‐1) provide additional insight into endothelial activation and vascular inflammation. Prior AD‐related studies have linked these markers to BBB permeability, vascular inflammation, amyloid and tau pathology, diagnostic status, and functional impairment [21, 22]. Importantly, circulating adhesion molecules are also strongly influenced by cardiometabolic burden and renal function, so any diagnosis‐associated difference must be evaluated against those determinants rather than attributed to AD biology by default.

Despite the growing literature on these individual biomarkers, an important gap remains. Most studies have examined astroglial, microglial, angiogenic, or endothelial markers separately, whereas AD biology is likely shaped by interactions among these systems. At the same time, previous endothelial biomarker research has remained highly heterogeneous and has emphasized the critical need for larger longitudinal analyses linking biomarker changes with clinical trajectories [23, 24].

The Alzheimer's Disease Neuroimaging Initiative (ADNI) provides a particularly appropriate platform for addressing this gap because it was designed as a longitudinal, multicenter biomarker resource for studying AD progression across the clinical continuum [25]. However, relatively few studies have jointly evaluated astroglial, microglial, angiogenic, and endothelial plasma biomarkers within the same ADNI‐based longitudinal framework.

In the present study, plasma GFAP, sTREM2, VEGF, sICAM‐1, and sVCAM‐1 were investigated in ADNI participants across the AD continuum. The study aimed to compare baseline biomarker distributions across diagnostic groups, evaluate longitudinal biomarker trajectories, and characterize the interrelationships among astroglial, microglial, angiogenic, and endothelial markers.

## 2 Materials and Methods

This study used data from the Alzheimer's Disease Neuroimaging Initiative (ADNI) database (www.adni.loni.usc.edu), which was launched in 2003 under the leadership of Principal Investigator Michael W. Weiner, MD. ADNI was designed to evaluate whether neuroimaging, biofluid biomarkers, and neuropsychological assessments can be integrated to monitor the progression of MCI and early AD.

### 2.1 Study Design and Data Source

The present analysis investigated plasma biomarkers related to the glial‐vascular axis across cognitively unimpaired participants, individuals with mild cognitive impairment, and patients with Alzheimer's disease dementia. The term cognitively unimpaired (CU) is used throughout in preference to cognitively normal.

The primary biomarker panel was selected a priori to represent astroglial activation, microglial‐related signaling, and vascular/endothelial dysfunction. Plasma glial fibrillary acidic protein was used as an astroglial marker; soluble triggering receptor expressed on myeloid cells 2 was used as a microglial‐related marker; and vascular endothelial growth factor, soluble intercellular adhesion molecule‐1, and soluble vascular cell adhesion molecule‐1 were used as vascular or endothelial‐related biomarkers. The R scripts used for data screening, preprocessing, statistical analysis, and figure generation are available at: https://github.com/Khodakaram/adni‐glial‐vascular‐axis.

### 2.2 Data Extraction and Cohort Construction

Clinical, demographic, genetic, cognitive, and biomarker files were downloaded from ADNI. Candidate files were screened using ADNI file descriptions to identify datasets containing participant identifiers, visit information, diagnostic variables, covariates, and target biomarker variables.

The clinical core dataset was constructed from ADNI diagnostic, demographic, cognitive, and genetic tables. Participant identifiers were harmonized using RID, and visit identifiers were standardized using available VISCODE, VISCODE2, examination‐date, or equivalent visit fields. Diagnosis was categorized as cognitively unimpaired, mild cognitive impairment, or Alzheimer's disease dementia, coded as CU, MCI, and AD, respectively. Baseline diagnosis was defined using the baseline visit when available. Visit‐level diagnosis was retained separately so that time‐varying diagnosis and conversion status could be modeled in sensitivity analyses. Time from baseline was calculated in years and used as the longitudinal time variable.

The primary covariates were baseline age, sex, education, and APOE ε4 carrier status. APOE ε4 status was coded as a binary variable indicating the presence or absence of at least one ε4 allele. Education was treated as a continuous variable, and sex was treated as a categorical variable. Age was entered as age at baseline and held constant within participants because age advances in exact proportion to time in study and cannot be separately identified from the time variable within an individual (Table S7).

### 2.3 Biomarker Harmonization and Transformation

Plasma GFAP data were provided by the ADNI Biomarker Core laboratory at the University of Pennsylvania, with the single molecule array (Simoa) Quanterix measure utilized as the primary GFAP variable [26]. Plasma sTREM2 data were generated collaboratively by the Cruchaga laboratory at Washington University in St. Louis (WashU) and the Haass laboratory at the Ludwig Maximilian University of Munich (LMU/DZNE); the corrected Meso Scale Discovery (MSD) sTREM2 measure was selected to represent this consensus data [27]. The vascular markers VEGF, sICAM‐1, and sVCAM‐1 were analyzed by the Myriad RBM laboratory using a Luminex multiplex platform, as part of the Foundation for the National Institutes of Health (FNIH) Biomarkers Consortium Plasma Proteomics Project [28].

Transformations were defined separately for each platform. The GFAP and sTREM2 releases report concentrations in pg/mL, and these were natural‐log transformed before modeling. The ADNI quality‐controlled multiplex release reports VEGF, sICAM‐1, and sVCAM‐1 as log10‐transformed values, notwithstanding that the column headers retain the original concentration units. This was verified against the corresponding raw multiplex release, where the correlation between each quality‐controlled value and log10 of the raw value was 1.000 with a regression slope of 1.000 for all three analytes. These variables were therefore converted to the natural log of concentration by multiplication by ln [10] rather than being log‐transformed a second time. All five biomarkers are consequently modeled on a natural‐log concentration scale, and regression coefficients are directly comparable across markers.

Regression coefficients from log‐transformed models were converted to approximate percentage differences using % Δ = [exp(*β*)−1] × 100. Only positive biomarker values were transformed; zero, negative, or missing values were treated as missing. Biomarker‐specific complete‐case datasets were used because assay availability differed across ADNI biomarker platforms. Participants were included in each model if they had the biomarker of interest, diagnosis, and complete required covariates. Missing biomarker values were not imputed.

### 2.4 Baseline Analysis

Baseline analyses were restricted to biomarker‐specific complete‐case analytic samples and to participants with a biomarker measurement at the baseline visit. Baseline biomarker distributions across CU, MCI, and AD groups were summarized descriptively and visualized using violin plots with embedded boxplots and individual data points.

Adjusted baseline associations were estimated by linear regression in which the natural log of the biomarker concentration was the dependent variable and diagnosis was the primary predictor, with CU as the reference category. Models were adjusted for baseline age, sex, education, and APOE ε4 carrier status and were fitted separately for each biomarker. The two reported contrasts are MCI versus CU and AD versus CU.

To distinguish statistical significance from discriminative relevance, the area under the receiver operating characteristic curve with 95% confidence intervals, Cohen's *d*, and the distribution overlap coefficient were computed for each biomarker and diagnostic contrast, both unadjusted and after residualization on the model covariates. The minimum AD‐versus‐CU difference detectable at 80% power was calculated for each panel from its residual standard deviation and group sizes.

### 2.5 Longitudinal Analysis

Longitudinal biomarker trajectories were evaluated using linear mixed‐effects models with participant‐level random intercepts to account for repeated measures within individuals. The main predictors were years from baseline, baseline diagnosis, and diagnosis‐by‐time interaction terms. CU was used as the reference diagnostic group. Models were adjusted for baseline age, sex, education, and APOE ε4 carrier status and were fitted separately for each plasma biomarker.

Participants without a biomarker measurement at the clinical baseline visit were included using later measurements. Consequently, the diagnosis terms in the mixed‐effects models are model‐estimated differences at time zero, extrapolated backwards for those participants, and are not equivalent to the observed baseline differences reported in Table 2. The proportion of each panel affected is reported in the Results and in Table S3.

The random‐effects structure was determined empirically rather than assumed. Because a substantial proportion of participants contributed a single measurement, a random slope could not be estimated on the full samples, and the comparison was therefore performed among participants with two or more measurements using likelihood ratio tests. A random intercept was retained for every biomarker on this basis; the vascular panel, which comprises exactly two visits per participant, cannot support a random slope at all. Residual distributions, non‐constant variance, and variance inflation factors were examined for every model.

Model‐estimated trajectories and 95% confidence intervals were generated from the fitted models. Each biomarker panel is plotted over the follow‐up range its own data support, up to the 95th percentile of observed follow‐up, so that the differing observation windows across assay platforms are explicit.

### 2.6 Correlation Analysis

Baseline glial‐vascular biomarker structure was assessed using pairwise Spearman correlations among the log‐transformed biomarkers. Pairwise complete observations were used for each biomarker pair. Confidence intervals were obtained by bootstrap resampling with 2000 replicates, and pairwise sample sizes are reported for every coefficient. Correlations based on fewer than 150 overlapping participants are identified as underpowered and exploratory.

### 2.7 Multiple‐Comparison Correction

*p*‐values were adjusted using the Benjamini–Hochberg false discovery rate procedure. Correction families were defined as follows: the 10 reported baseline diagnostic contrasts (two contrasts for each of five biomarkers); the 25 reported longitudinal terms (five terms for each of five biomarkers); and the 10 pairwise correlations. Covariate and intercept terms were not included in any correction family. Because the choice of family can affect borderline results, *q*‐values were additionally computed under three alternative definitions: within each biomarker separately, globally across all 35 reported model terms, and across every estimated coefficient. Results of this sensitivity analysis are reported in Table S6.

### 2.8 Vascular Risk Factor Adjustment

Because circulating adhesion molecules and growth factors are influenced by cardiometabolic burden and renal function independently of AD status, the vascular models were refitted with additional covariates. Body mass index and systolic blood pressure were derived from ADNI vital signs; antihypertensive and antidiabetic medication use from concomitant medication records; and smoking history from medical history records. Estimated glomerular filtration rate was calculated from serum creatinine using the 2021 CKD‐EPI equation [29]. Creatinine was available for 554 of the 566 participants in the vascular panel.

Models were fitted in four tiers of increasing adjustment, each on its own complete‐case sample, with sample size reported at every tier so that the cost of adjustment is visible. Unadjusted and adjusted estimates are presented side by side in Tables S23 and S24.

### 2.9 Sensitivity Analyses

Six sensitivity analyses were performed. First, longitudinal models were refitted among participants with two or more measurements. Second, a within‐between decomposition separated within‐person change from between‐person differences. Third, models were refitted among participants with at least 2 years of follow‐up where the data permitted. Fourth, diagnosis was modeled as a time‐varying variable, and separately the MCI group was split into stable MCI and MCI‐to‐AD converters. Fifth, informative dropout was assessed by modeling whether the baseline biomarker value predicted return for a second measurement. Sixth, assay quality flags from the raw multiplex release were used to exclude samples below the detection limit or flagged as outliers.

All analyses were performed in R (version 4.5.0) using the lme4 and lmerTest packages for mixed‐effects models.

## 3 Results

### 3.1 Cohort Construction and Analytic Samples

The ADNI clinical core dataset included 3788 participants. After excluding individuals without a baseline visit, without a baseline CU/MCI/AD diagnosis, or with incomplete required baseline covariates, 2650 participants remained in the covariate‐complete clinical cohort, including 1027 CU, 1187 MCI, and 436 ad participants. Because the plasma biomarkers were derived from different ADNI assay platforms, analytic sample sizes differed by biomarker. Baseline complete‐case models included 832 participants for GFAP, 1001 for sTREM2, and 566 for VEGF, sICAM‐1, and sVCAM‐1. Longitudinal models included 1252 participants and 1671 observations for GFAP, 1031 participants and 1771 observations for sTREM2, and 566 participants with 1061 observations for the vascular markers. Cohort construction is summarized in Figure S1.

The three panels differ markedly in longitudinal density (Tables S1 and S2). A single measurement was contributed by 69.7% of CU, 68.8% of MCI, and 69.1% of AD participants in the GFAP panel, and by 54.9%, 52.3%, and 83.6%, respectively, in the sTREM2 panel. The vascular panel consists of exactly two visits, baseline and month 12, available for 93.1% of CU, 87.1% of MCI, and 85.7% of AD participants, with a median follow‐up of 1.0 year and a maximum observed span of 1.63 years. Median follow‐up was 0.00 years for GFAP and sTREM2, with third quartiles of 0.98 to 2.05 years and maxima of 19.25 and 9.37 years, respectively.

Longitudinal samples exceed baseline samples for two panels because participants without a baseline biomarker measurement were included using later measurements. For GFAP, 421 of 1252 participants (33.6%) had no baseline measurement, and among these the first available measurement occurred a median of 4.07 years after baseline; for sTREM2 the figure was 30 of 1031 (2.9%), and for the vascular panel none (Table S3).

Assay availability was not random with respect to diagnosis, and the direction of selection differs between panels (Tables S4 and S5). Cognitively unimpaired participants constitute 53.6% of the GFAP sample but only 10.2% of the vascular sample, compared with 35.0% and 46.9% respectively among participants excluded from each (*p* = 1.2 × 10^−19^ and *p* = 8.4 × 10^−59^). The vascular sample is also, on average, 3 years older, 15 percentage points less female, and eight points more frequently APOE ε4 positive than participants excluded from it.

### 3.2 Baseline Biomarker Distributions

**FIGURE 1:**
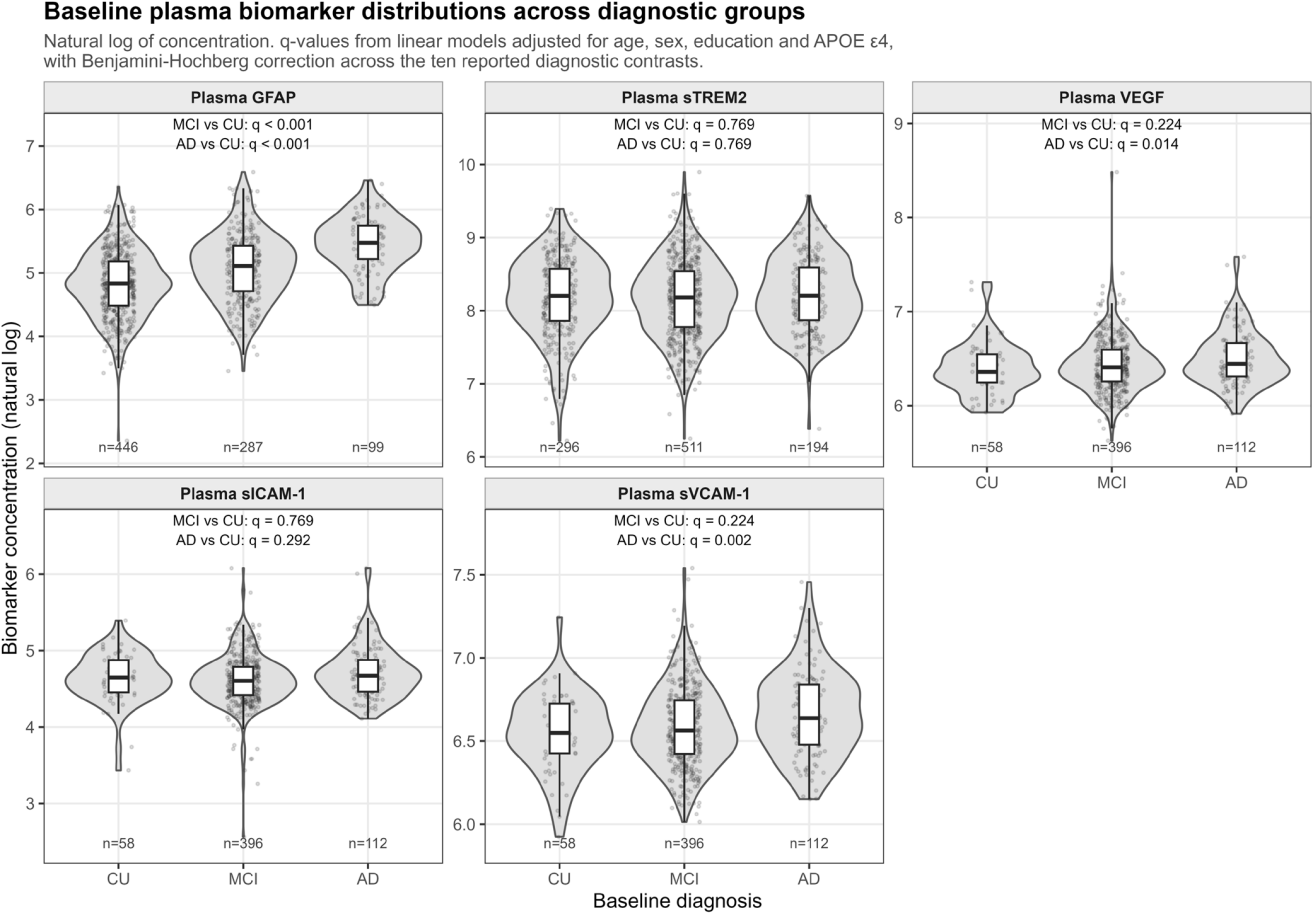
Baseline plasma biomarker distributions across diagnostic groups. Baseline distributions of plasma GFAP, sTREM2, VEGF, sICAM‐1, and sVCAM‐1 across CU, MCI, and AD groups, using the biomarker‐specific complete‐case analytic samples of the adjusted baseline regression models. All markers are shown on the natural log of concentration, so panels are directly comparable. Violin plots show distribution density, embedded boxplots show median and interquartile range, and points represent individual participants. Sample sizes below each group correspond to the complete‐case analytic sample for that biomarker. *Q*‐values above each panel derive from linear regression models adjusted for baseline age, sex, education, and APOE ε4 status, with Benjamini–Hochberg correction across the 10 reported diagnostic contrasts.

Baseline descriptive distributions showed a clear diagnostic gradient for plasma GFAP, with median values of 125.70, 165.80, and 238.6 pg/mL in CU, MCI, and AD, respectively. sTREM2 distributions were broadly overlapping, with medians of 3647, 3571, and 3656 pg/mL. Among the vascular markers, VEGF rose from 577.0 to 606.5 to 629.5 pg/mL and sVCAM‐1 from 699.0 to 709.0 to 763.5 ng/mL across CU, MCI, and AD, while sICAM‐1 showed no ordered separation (104.5, 100.0, and 107.0 ng/mL). Group‐specific medians and interquartile ranges are shown in Table 1, and distributions in Figure 1.

**TABLE 1:** Baseline plasma biomarker concentrations by diagnostic group.

Biomarker	Unit	CU	MCI	AD
Plasma GFAP (Simoa)	pg/mL	125.7 [88.7–178.0] *n* = 446	165.8 [111.4–228.1] *n* = 287	238.6 [184.6–312.9] *n* = 99
Plasma sTREM2 (MSD)	pg/mL	3647.4 [2594.4–5296.1] *n* = 296	3570.7 [2386.8–5124.8] *n* = 511	3655.6 [2616.6–5391.5] *n* = 194
Plasma VEGF (Myriad RBM)	pg/mL	577.0 [516.0–695.8] *n* = 58	606.5 [521.8–731.3] *n* = 396	629.5 [550.0–785.3] *n* = 112
Plasma sICAM‐1 (Myriad RBM)	ng/mL	104.5 [86.0–131.0] *n* = 58	100.0 [83.0–120.0] *n* = 396	107.0 [86.8–131.2] *n* = 112
Plasma sVCAM‐1 (Myriad RBM)	ng/mL	699.0 [617.8–832.8] *n* = 58	709.0 [616.0–849.5] *n* = 396	763.5 [650.7–933.7] *n* = 112

*Note:* Values are median [interquartile range] in native concentration units. Sample sizes correspond to the biomarker‐specific complete‐case baseline analytic samples. VEGF, sICAM‐1, and sVCAM‐1 are reported after back‐transformation from the log_10_ scale used in the ADNI quality‐controlled multiplex release.

Abbreviations: AD, Alzheimer's disease dementia; CU, cognitively unimpaired; MCI, mild cognitive impairment.

### 3.3 Adjusted Baseline Diagnostic Associations

After adjustment for baseline age, sex, education, and APOE ε4 status, GFAP remained strongly associated with diagnostic status. Compared with CU, MCI was associated with a 21.5% higher GFAP level (95% CI 13.1 to 30.6; *q* = 7.0 × 10^−7^) and AD with a 56.3% higher level (95% CI 40.4 to 74.2; *q* = 1.7 × 10^−14^). sTREM2 was not significantly associated with baseline diagnosis.

Among the vascular markers, VEGF was 14.1% higher in AD than CU (95% CI 4.0 to 25.3; *q* = 0.014) and sVCAM‐1 was 14.7% higher (95% CI 6.1 to 24.0; *q* = 0.002). Neither MCI contrast was significant, and sICAM‐1 was not significantly associated with diagnosis in either contrast. Adjusted baseline associations are summarized in Table 2.

**TABLE 2:** Adjusted baseline associations between plasma biomarkers and diagnostic group.

Biomarker	Contrast	*β* (SE)	% difference (95% CI)	*p*	*q* (FDR)	*n*
Plasma GFAP (Simoa)	MCI vs. CU	0.195 (0.037)	21.5 (13.1 to 30.6)	1.39 × 10^−7^	6.97 × 10^−7^	832
	AD vs. CU	0.447 (0.055)	56.3 (40.4 to 74.2)	1.66 × 10^−15^	1.66 × 10^−14^	832
Plasma sTREM2 (MSD)	MCI vs. CU	0.011 (0.039)	1.2 (−6.3 to 9.2)	0.769	0.769	1001
	AD vs. CU	0.022 (0.050)	2.2 (−7.4 to 12.8)	0.666	0.769	1001
Plasma VEGF (Myriad RBM)	MCI vs. CU	0.064 (0.041)	6.6 (−1.6 to 15.5)	0.119	0.224	566
	AD vs. CU	0.132 (0.048)	14.1 (4.0 to 25.3)	0.0056	0.014	566
Plasma sICAM‐1 (Myriad RBM)	MCI vs. CU	−0.017 (0.049)	−1.7 (−10.8 to 8.3)	0.733	0.769	566
	AD vs. CU	0.073 (0.058)	7.6 (−3.9 to 20.4)	0.204	0.292	566
Plasma sVCAM‐1 (Myriad RBM)	MCI vs. CU	0.051 (0.034)	5.2 (−1.6 to 12.5)	0.134	0.224	566
	AD vs. CU	0.137 (0.040)	14.7 (6.1 to 24.0)	5.75 × 10^−4^	0.0019	566

*Note:* Linear regression models with the natural log of biomarker concentration as the dependent variable, adjusted for baseline age, sex, education, and APOE ε4 carrier status. CU is the reference category. Percentage differences were derived as [exp(*β*)−1] × 100. Benjamini–Hochberg correction was applied across the 10 reported diagnostic contrasts. Table S6 reports *q*‐values under three alternative correction families; no result changes significance status at *q* < 0.05.

The statistical significance of these differences should not be read as diagnostic utility. For AD versus CU, plasma GFAP achieved an area under the receiver operating characteristic curve of 0.822 (95% CI 0.779 to 0.866), with a Cohen's *d* of 1.23 and a distribution overlap coefficient of 0.51. The corresponding areas under the curve were 0.625 for sVCAM‐1, 0.612 for VEGF, 0.528 for sICAM‐1, and 0.507 for sTREM2, with overlap coefficients between 0.83 and 0.92 (Table S11). The vascular differences therefore represent group‐level shifts against a background of substantial individual overlap.

The two null baseline findings are power‐limited rather than clean negatives. The minimum AD‐versus‐CU difference detectable at 80% power was 16.5% for sICAM‐1 against an observed 7.6% and 14.3% for sTREM2 against an observed 2.2% (Table S12). Neither result excludes effects smaller than approximately 15%.

Neither diagnosis‐by‐APOE ε4 nor diagnosis‐by‐sex interactions were significant for any biomarker after correction; the strongest signal was a diagnosis‐by‐APOE ε4 interaction for VEGF (nominal *p* = 0.14, *q* = 0.92) (Table S14).

### 3.4 Longitudinal Biomarker Trajectories

Plasma GFAP increased over time in the CU reference group by an estimated 4.50% per year (95% CI 3.79 to 5.21; *q* = 1.0 × 10^−33^). Baseline GFAP levels were substantially higher in MCI and AD than CU, but neither diagnosis‐by‐time interaction was significant, indicating that the rate of GFAP change did not differ materially by baseline diagnosis. Plasma sTREM2 also increased over time in CU participants, by 2.99% per year (95% CI 1.56 to 4.44; *q* = 1.2 × 10^−4^), with no significant baseline diagnostic differences and no FDR‐significant diagnosis‐by‐time interactions.

**FIGURE 2:**
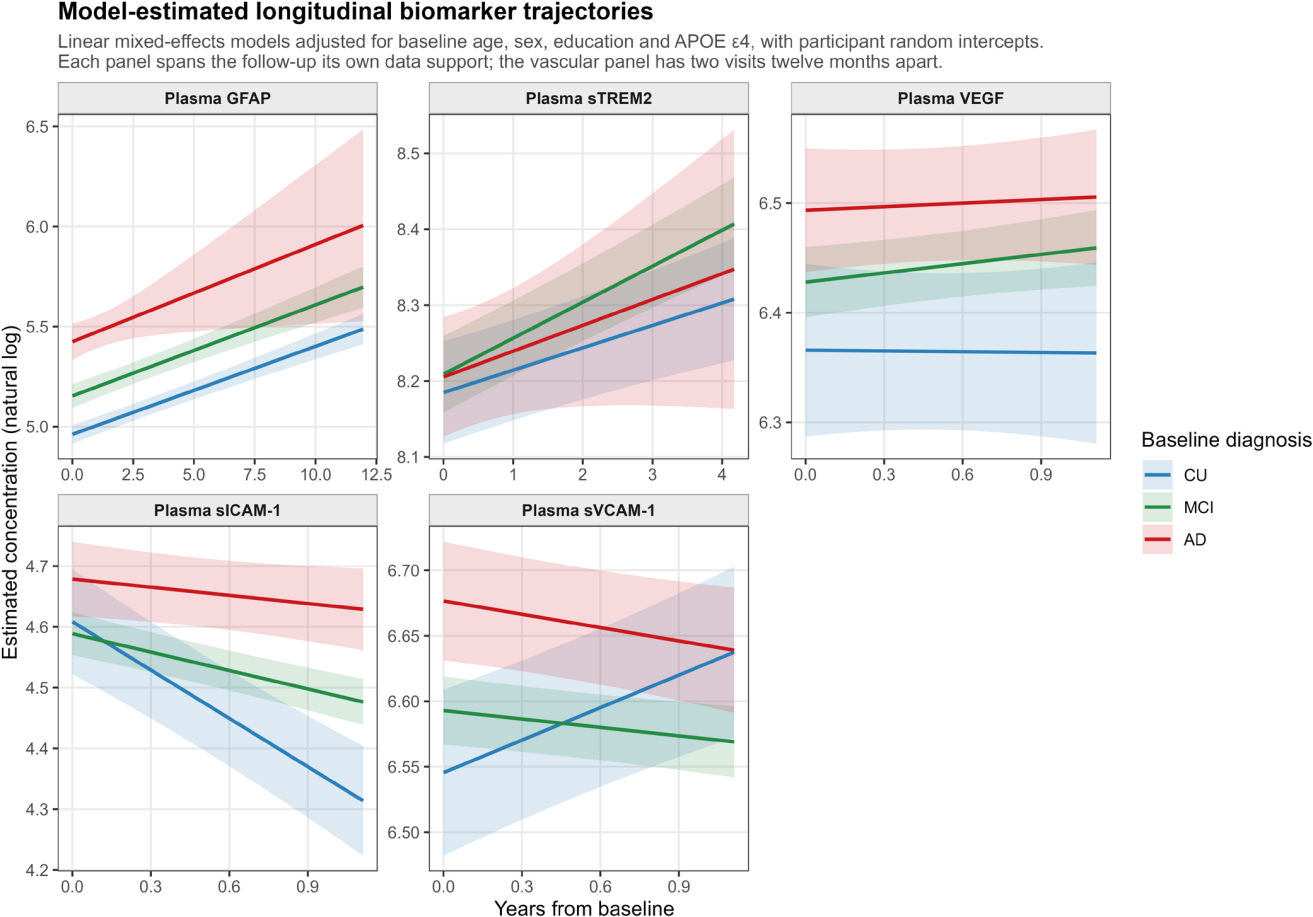
Model‐estimated longitudinal biomarker trajectories. Trajectories of plasma GFAP, sTREM2, VEGF, sICAM‐1, and sVCAM‐1 according to baseline diagnosis, from linear mixed‐effects models adjusted for baseline age, sex, education, and APOE ε4 status, with participant‐level random intercepts. Lines represent fitted mean biomarker levels and shaded ribbons represent 95% confidence intervals. Each panel spans only the follow‐up range its own data support, up to the 95th percentile of observed follow‐up; the vascular panel comprises two visits 12 months apart, with a maximum observed span of 1.63 years.

VEGF showed an AD‐associated baseline elevation consistent with the cross‐sectional models but no significant time effect or diagnosis‐by‐time interaction. The endothelial adhesion markers showed the clearest diagnosis‐dependent patterns over the 12‐month vascular observation window. sICAM‐1 declined in CU participants by 23.3% per year (95% CI −28.7 to −17.5; *q* = 3.0 × 10^−11^), and this decline was attenuated in MCI and AD, with positive diagnosis‐by‐time interactions of 17.8% (*q* = 1.4 × 10^−4^) and 24.6% (*q* = 1.3 × 10^−5^). Conversely, sVCAM‐1 increased in CU participants by 8.6% per year (*q* = 1.5 × 10^−4^) with negative diagnosis‐by‐time interactions of −9.9% (*q* = 1.3 × 10^−5^) and −11.0% (*q* = 2.4 × 10^−5^). Longitudinal estimates are summarized in Table 3 and trajectories in Figure 2.

**TABLE 3:** Linear mixed‐effects model estimates of longitudinal biomarker trajectories.

Biomarker	Effect	*β* (SE)	% change (95% CI)	*p*	*q* (FDR)
Plasma GFAP (Simoa) 1671 obs/1252 participants	Time, years (CU slope)	0.044 (0.003)	4.50 (3.79 to 5.21)	4.04 × 10^−35^	1.01 × 10^−33^
	MCI vs. CU at baseline	0.192 (0.034)	21.2 (13.4 to 29.5)	1.87 × 10^−8^	1.17 × 10^−7^
	AD vs. CU at baseline	0.463 (0.049)	58.9 (44.2 to 75.1)	2.54 × 10^−20^	3.17 × 10^−19^
	Time × MCI	0.002 (0.006)	0.15 (−1.01 to 1.32)	0.799	0.880
	Time × AD	0.005 (0.022)	0.46 (−3.81 to 4.92)	0.835	0.880
Plasma sTREM2 (MSD) 1771 obs/1031 participants	Time, years (CU slope)	0.029 (0.007)	2.99 (1.56 to 4.44)	3.90 × 10^−5^	1.22 × 10^−4^
	MCI vs. CU at baseline	0.024 (0.038)	2.5 (−4.9 to 10.3)	0.521	0.724
	AD vs. CU at baseline	0.021 (0.049)	2.1 (−7.3 to 12.4)	0.677	0.846
	Time × MCI	0.018 (0.009)	1.81 (0.03 to 3.62)	0.047	0.091
	Time × AD	0.005 (0.023)	0.45 (−4.00 to 5.12)	0.845	0.880
Plasma VEGF (Myriad RBM) 1061 obs/566 participants	Time, years (CU slope)	−0.002 (0.034)	−0.23 (−6.72 to 6.72)	0.947	0.947
	MCI vs. CU at baseline	0.062 (0.041)	6.4 (−1.9 to 15.4)	0.135	0.241
	AD vs. CU at baseline	0.128 (0.048)	13.6 (3.4 to 24.9)	0.0081	0.017
	Time × MCI	0.030 (0.037)	3.09 (−4.11 to 10.84)	0.411	0.604
	Time × AD	0.013 (0.043)	1.31 (−6.89 to 10.23)	0.763	0.880
Plasma sICAM‐1 (Myriad RBM) 1061 obs/566 participants	Time, years (CU slope)	−0.265 (0.037)	−23.29 (−28.69 to −17.49)	3.66 × 10^−12^	3.05 × 10^−11^
	MCI vs. CU at baseline	−0.020 (0.045)	−2.0 (−10.3 to 7.1)	0.663	0.846
	AD vs. CU at baseline	0.070 (0.053)	7.3 (−3.2 to 18.9)	0.182	0.284
	Time × MCI	0.164 (0.040)	17.84 (8.93 to 27.47)	4.93 × 10^−5^	1.37 × 10^−4^
	Time × AD	0.220 (0.047)	24.65 (13.74 to 36.59)	3.09 × 10^−6^	1.29 × 10^−5^
Plasma sVCAM‐1 (Myriad RBM) 1061 obs/566 participants	Time, years (CU slope)	0.083 (0.020)	8.64 (4.37 to 13.07)	5.80 × 10^−5^	1.45 × 10^−4^
	MCI vs. CU at baseline	0.047 (0.033)	4.9 (−1.7 to 11.9)	0.154	0.256
	AD vs. CU at baseline	0.131 (0.039)	14.0 (5.7 to 23.0)	7.14 × 10^−4^	0.0016
	Time × MCI	−0.104 (0.022)	−9.91 (−13.71 to −5.94)	2.75 × 10^−6^	1.29 × 10^−5^
	Time × AD	−0.117 (0.026)	−11.02 (−15.38 to −6.43)	6.69 × 10^−6^	2.39 × 10^−5^

*Note:* Linear mixed‐effects models with the natural log of biomarker concentration as the dependent variable, participant‐level random intercepts, and fixed effects for years from baseline, baseline diagnosis, and their interaction, adjusted for baseline age, sex, education, and APOE ε4 carrier status. CU is the reference category. Baseline age is held constant within participants because age advances in exact proportion to time in study. Percentage changes were derived as [exp(*β*)−1] × 100 and are expressed per year. Benjamini–Hochberg correction was applied across the 25 reported longitudinal terms. Diagnosis terms are model‐estimated differences at time zero; for GFAP, 33.6% of participants had no baseline measurement, so these are partly extrapolated and are not equivalent to the observed baseline differences in Table 2. The vascular panel comprises two visits 12 months apart, with a maximum observed span of 1.63 years, so its time and interaction terms describe a 12‐month change contrast.

Because a majority of GFAP and sTREM2 participants contributed a single measurement, the GFAP time effect was examined further. It was 4.50% per year in the full sample, 5.74% among the 384 participants with two or more measurements, 5.87% among the 294 followed for at least 2 years, and 6.08% as a pure within‐person estimate in a within‐between decomposition (Tables S15–S17). Individual within‐person trajectories are shown in Figure S3. The GFAP finding is therefore not an artifact of participants observed only once.

### 3.5 Sensitivity Analyses

Overall, 25.0% of participants with baseline MCI converted to AD during follow‐up, and 11.3% of cognitively unimpaired participants progressed; conversion rates within the analytic panels were 29.8% for GFAP, 41.3% for sTREM2, and 54.0% for the vascular panel (Table S18). Modeling diagnosis as a time‐varying variable reproduced all findings (Table S19). Separating stable MCI from MCI‐to‐AD converters likewise reproduced them: the sICAM‐1 time interaction was 20.2% per year for stable MCI and 16.1% for converters, and the sVCAM‐1 interaction was −9.7% and −10.0%, respectively (Table S20). Conversion heterogeneity therefore does not generate the observed pattern.

Attrition in the vascular panel was modest and only slightly differential (93.1%, 87.1%, and 85.7% retention in CU, MCI, and AD). Baseline GFAP did not predict return for a second measurement. For sTREM2, AD participants were substantially less likely to return (odds ratio 0.25, *p* = 3.5 × 10^−9^), and for sICAM‐1 and VEGF, higher baseline values predicted non‐return (odds ratios 0.45 and 0.34, *p* = 0.036 and 0.013) (Table S21). Assay quality flags affected between 0% and 0.4% of vascular samples, were balanced between the two visits, and their exclusion left all estimates unchanged (Table S22).

Adjusting the vascular models for body mass index, systolic blood pressure, antihypertensive and antidiabetic medication use, smoking history, and estimated glomerular filtration rate left the longitudinal findings essentially unchanged. Across the unadjusted and fully adjusted models, the sICAM‐1 diagnosis‐by‐time interactions moved from 17.8% to 17.6% and from 24.6% to 24.8%, and the sVCAM‐1 interactions from −9.9% to −9.7% and from −11.0% to −11.0%, all remaining significant at *p* < 10^−4^ on 550 participants. Baseline associations attenuated modestly but persisted: the AD‐versus‐CU difference fell from 14.1% to 12.4% for VEGF (*p* = 0.012) and from 14.7% to 12.3% for sVCAM‐1 (*p* = 0.004). sICAM‐1 showed the greatest attenuation, from 7.6% to 4.5%, and was the most sensitive of the three markers to renal adjustment, although it was not significantly associated with diagnosis in either specification. Vascular risk factors were closely balanced across diagnostic groups, which accounts for the small movement in estimates (Tables S23–S26).

*Q*‐values were robust to the definition of the correction family. Across four family definitions, the VEGF AD‐versus‐CU association ranged from *q* = 0.010 to 0.013, and the sVCAM‐1 association from *q* = 0.001 to 0.002, and no result changed significance status at *q* < 0.05 (Table S6). Model diagnostics are reported in Tables S8–S10 and Figure S2. Residuals were approximately symmetric for four of the five biomarkers; VEGF baseline residuals were right‐skewed with evidence of non‐constant variance (*p* = 0.004), and heteroscedasticity‐consistent standard errors did not alter the VEGF conclusions.

### 3.6 Baseline Glial‐Vascular Correlation Structure

Because the five plasma biomarkers were generated from different ADNI assay platforms, overlap between biomarker datasets varied substantially, and correlations were therefore computed on pairwise complete observations. Correlations among the vascular markers were based on the full vascular sample and were well estimated: VEGF correlated with sICAM‐1 at *ρ* = 0.31 (95% CI 0.23 to 0.38) and with sVCAM‐1 at *ρ* = 0.30 (95% CI 0.22 to 0.37), and sICAM‐1 with sVCAM‐1 at *ρ* = 0.25 (95% CI 0.17 to 0.33), all on *n* = 566 and all significant after correction.

**FIGURE 3:**
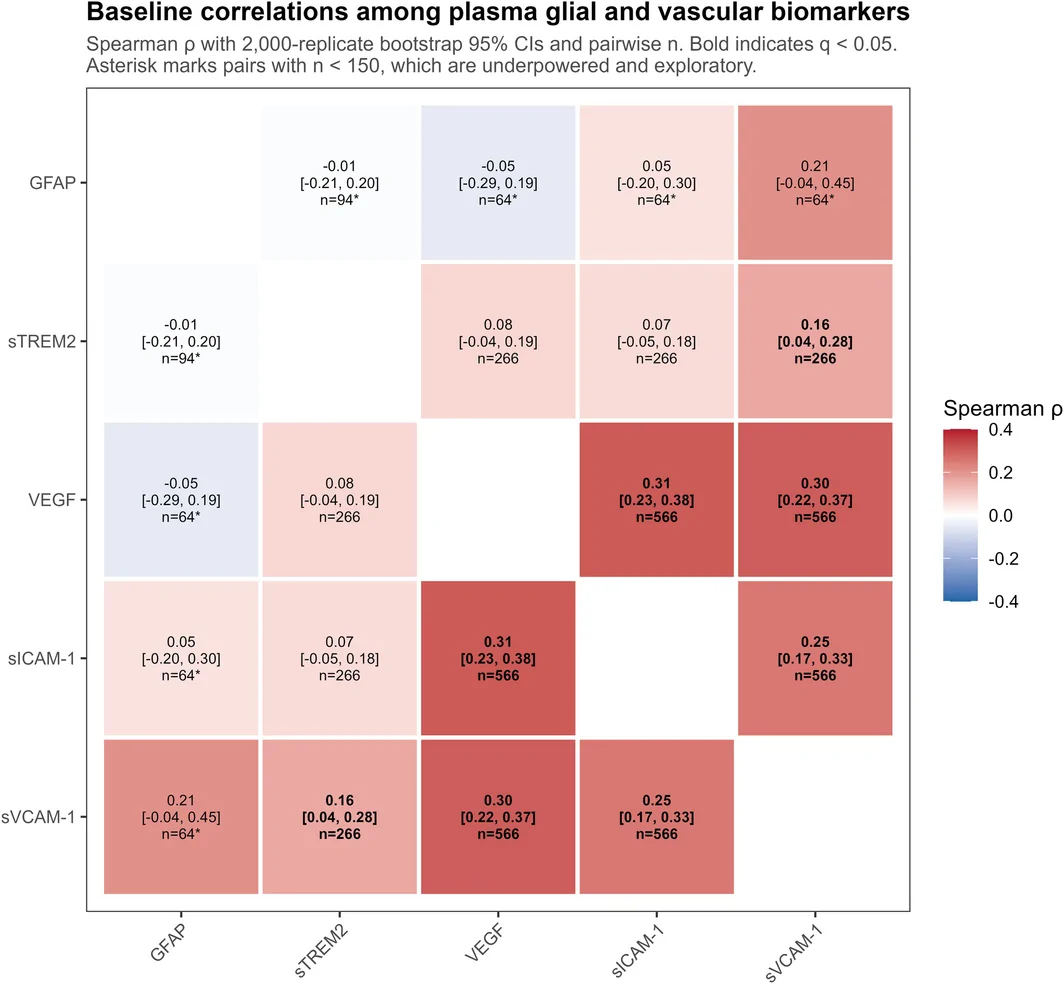
Baseline glial‐vascular biomarker correlation heatmap. Pairwise Spearman correlations among log‐transformed baseline plasma GFAP, sTREM2, VEGF, sICAM‐1, and sVCAM‐1. Tile color indicates Spearman's *ρ*. Cell labels show the coefficient, a 2000‐replicate bootstrap 95% confidence interval, and the pairwise sample size. Bold values are significant at *q* < 0.05 after Benjamini–Hochberg correction across the 10 pairwise comparisons. Asterisks mark pairs based on fewer than 150 overlapping participants, which are underpowered and exploratory; smaller pairwise n reflects limited overlap between assay platforms rather than additional diagnostic exclusions.

Correlations involving GFAP were based on 64 to 94 overlapping participants and were not informative. GFAP correlated with sVCAM‐1 at *ρ* = 0.21 with a 95% confidence interval of −0.04 to 0.45 (*n* = 64, *q* = 0.28), and with sTREM2 at *ρ* = −0.01 (95% CI −0.21 to 0.20, *n* = 94). Neither is statistically significant, and the confidence intervals are wide enough to encompass both null and moderate associations. sTREM2 correlated weakly with sVCAM‐1 (*ρ* = 0.16, 95% CI 0.04 to 0.28, *n* = 266). The correlation structure therefore supports coupling among the circulating vascular markers, but the present data cannot determine whether the glial and vascular domains are separable. The correlation matrix is shown in Figure 3 and Table S13.

## 4 Discussion and Conclusion

The present study supports a pattern of temporal heterogeneity among glial and vascular plasma biomarkers across the AD clinical continuum. Plasma GFAP showed the strongest and most consistent diagnosis‐associated elevation, with sustained separation between diagnostic groups in longitudinal models and the only area under the curve of clinical interest. Plasma sTREM2 showed overlapping baseline profiles across diagnostic groups but a significant annual increase in cognitively unimpaired participants. Vascular adhesion markers showed diagnosis‐dependent changes over a 12‐month interval. Together, these observations suggest that glial and vascular plasma biomarkers may capture partially distinct biological processes rather than a single synchronized disease trajectory.

Astrocytic reactivity is one of the earliest and most robust measurable biological responses to amyloid‐beta oligomerization and initial plaque deposition. Plasma GFAP discriminates cognitively unimpaired aging from mild cognitive impairment and AD dementia, with changes often preceding or accelerating faster than other plasma markers [30, 31]. The estimate of a 4.50% annual increase in cognitively unimpaired participants, rising to 6.08% when restricted to within‐person change, is consistent with that literature and is stable across every sensitivity analysis performed.

In contrast to the robust astrocytic signal, sTREM2 presents a more complex profile. CSF sTREM2 is a valuable longitudinal marker of central neuroinflammation that often peaks around early symptomatic stages and is strongly associated with tau‐related processes and neuronal injury [13, 14]. Peripheral plasma sTREM2 trajectories frequently fail to mirror these central dynamics reliably, remaining largely static or overlapping across clinical stages [15, 16]. The data are consistent with that picture cross‐sectionally, although longitudinal patterns may be more stage‐dependent. In the present study, a modest annual increase was detected among cognitively unimpaired participants. The null baseline finding for sTREM2 should be interpreted cautiously, as the analysis was limited by statistical power and does not exclude diagnostic differences smaller than approximately 14%.

A significant gap in the literature regarding vascular biomarkers is the frequent discordance between cross‐sectional and longitudinal findings. While cross‐sectional studies have linked elevated endothelial dysfunction biomarkers to concurrent cognitive impairment [21], sICAM‐1 declined over 12 months in cognitively unimpaired participants with an attenuated decline in MCI and AD, whereas sVCAM‐1 increased in cognitively unimpaired participants with negative diagnosis‐by‐time interactions. These divergent patterns were robust to adjustment for cardiometabolic burden and renal function, to time‐varying diagnosis, to separation of stable MCI from converters, and to exclusion of assay‐flagged samples.

Nevertheless, the biological interpretation of this result requires caution for three reasons. First, the observation window was limited to 12 months, with a maximum observed follow‐up duration of 1.63 years, and no participant in the vascular panel reached 2 years of follow‐up. An interval of this length cannot support inference about trajectories across a prodromal period that may extend over decades; therefore, these findings are described as a 12‐month change contrast rather than as evidence of stage‐dependent regulation. Second, the magnitude of the sICAM‐1 decline in cognitively unimpaired participants is high for a stable population, and the ADNI multiplex release carries no plate or assay‐run identifiers, so a run‐to‐run analytical effect can be neither confirmed nor excluded. Third, the cognitively unimpaired reference group in this panel comprises only 58 participants. Replication in a cohort with longer follow‐up, a larger unimpaired reference group, and documented assay batch structure is required before these trajectories can be attributed to vascular biology.

Our finding of a VEGF elevation in AD relative to cognitively unimpaired participants requires reconciliation with the meta‐analysis of Xu et al., which concluded that VEGF is an effective biomarker for vascular dementia but not for AD [20]. Three considerations apply. Our VEGF values derive from a multiplex bead‐based platform, whereas the studies aggregated in that meta‐analysis predominantly used single‐analyte immunoassays, and absolute concentrations differ substantially between platforms. Our vascular subsample is an ADNI‐1 subset that is 70% MCI and only 10% cognitively unimpaired, older, and more frequently APOE ε4 positive than excluded participants, and is therefore not a representative diagnostic cross‐section. Most importantly, the two findings are not in fact in conflict. A 14.1% group‐level difference corresponding to an area under the curve of 0.61 with 83% distribution overlap, attenuating to 12.4% after renal adjustment, is entirely compatible with the conclusion that VEGF is not an effective diagnostic biomarker at the individual level.

The same distinction applies to the adhesion molecules. While the diagnosis‐associated differences in sVCAM‐1 are statistically robust and survive full covariate adjustment, the corresponding area under the curve of 0.63 and distribution overlap of 0.83 indicate no individual‐level discriminative value. These markers should be understood as group‐level evidence of endothelial involvement in AD rather than as candidate diagnostic biomarkers, and no claim is made that they are comparable to GFAP in this respect.

A related question is whether glial and vascular processes interact. Singh et al. recently reported a significant VCAM‐1 by GFAP interaction in relation to cognitive performance in Black adults in the ARCHES study, a finding directly relevant to the premise that these domains should be studied jointly [32]. Our data can neither support nor refute it. GFAP and the vascular markers were assayed in largely non‐overlapping ADNI subsamples, leaving only 64 participants with both GFAP and sVCAM‐1 at baseline, in whom the correlation is 0.21 with a confidence interval spanning −0.04 to 0.45. The weak glial‐vascular correlations were initially interpreted as evidence of partly separable biological domains; however, when considered alongside the confidence intervals, this interpretation is not supported, and the question remains open. Testing a joint glial‐vascular relationship against cognitive outcomes requires a cohort in which both analyte classes are measured in the same participants, and this represents an important priority for future research.

Several limitations warrant emphasis. Direct cross‐analyte kinetic comparisons face substantial analytical variance because data are generated across fundamentally diverse detection technologies: sub‐picogram digital counting for GFAP, electrochemiluminescence for sTREM2, and bead‐based multiplexing for vascular markers [33, 34]. Circulating plasma markers serve as indirect systemic proxies and lack the spatial resolution required to map localized neurodegeneration. The use of biomarker‐specific complete‐case samples means that models were not fitted in identical participant sets, and selection into the panels was strongly non‐random and, critically, ran in opposite directions: the GFAP sample is enriched for cognitively unimpaired participants while the vascular sample is depleted of them. Any survivorship‐type bias would therefore act in opposite directions for the glial and vascular findings, and the two sets of results should not be assumed to carry the same selection artifact.

The longitudinal inference is also unevenly supported across panels. Approximately 69% of GFAP participants and between 52% and 84% of sTREM2 participants contributed a single measurement; therefore, the estimated time effects for these markers are derived from a minority of participants with repeat sampling. This limitation was addressed through within‐between decomposition and restriction analyses, which reproduced the GFAP result. Additionally, one third of the GFAP longitudinal sample lacked a baseline measurement, meaning that the model‐estimated diagnosis differences at time zero are partly extrapolated. Informative dropout represents a further concern, particularly for sTREM2, for which participants with AD were markedly less likely to return for a second measurement.

In conclusion, the AD clinical continuum was characterized by heterogeneous glial and vascular plasma biomarker trajectories. Plasma GFAP showed the strongest and most consistent diagnosis‐associated elevation and the only discriminative performance of potential clinical relevance, supporting its value as a robust marker of astroglial activation across diagnostic groups. Plasma sTREM2 showed overlapping cross‐sectional profiles with a modest longitudinal increase. sICAM‐1 and sVCAM‐1 showed divergent 12‐month changes by diagnostic group that were robust to extensive sensitivity analysis, but that require replication over longer follow‐up and with documented assay batch structure before they can be interpreted as stage‐dependent vascular regulation. Future studies integrating longitudinal plasma biomarkers with CSF measures, neuroimaging, vascular assessments, and clinical outcomes, and measuring glial and vascular analytes in the same participants are needed to determine whether these systemic patterns correspond to localized neurovascular injury.

## Author Contributions

K.J. designed the research; acquired, curated, and harmonized the ADNI data; developed and implemented the data‐screening, preprocessing, statistical‐analysis, and figure‐generation scripts; analyzed the data; interpreted the results; prepared the tables and figures; and wrote, revised, and approved the manuscript. Data used in preparation of this article were obtained from the Alzheimer's Disease Neuroimaging Initiative (ADNI) database. The investigators within ADNI contributed to the design and implementation of ADNI and/or provided data but did not participate in the analysis, interpretation, or writing of this report.

## Funding

The authors have nothing to report.

## Ethics Statement

This study was conducted using ADNI data. The ADNI study is ethically approved and operated in accordance with the Declaration of Helsinki, 1964.

## Conflicts of Interest

The authors declare no conflicts of interest.

## Supporting information


**Figure S1:** Study flow and biomarker‐specific analytic cohorts. Flow diagram showing construction of the ADNI clinical core cohort and the biomarker‐specific analytic samples. Participants were excluded for missing baseline visit, missing baseline CU/MCI/AD diagnosis, or incomplete required baseline covariates. Baseline and longitudinal model sample sizes are shown separately for each biomarker. CU, cognitively unimpaired; MCI, mild cognitive impairment; AD, Alzheimer's disease dementia; APOE4, apolipoprotein E ε4 carrier status.
**Figure S2:** Normal quantile plots of baseline model residuals. Residuals from the covariate‐adjusted baseline linear regression model for each biomarker, plotted against theoretical normal quantiles. Points falling along the reference line indicate approximate normality. VEGF residuals depart from the line in the upper tail, consistent with the right skew and non‐constant variance reported in Table S9.
**Figure S3:** Within‐person plasma GFAP trajectories. Individual trajectories for participants contributing two or more GFAP measurements (*n* = 384). Gray lines represent individual participants; colored lines and shaded ribbons show fitted group trends with 95% confidence intervals by baseline diagnosis. The within‐person increase is 6.08% per year, larger than the 4.50% obtained from the pooled model that also includes single‐measurement participants.

**Table S1:** Number of biomarker measurements per participant, by panel and diagnostic group.
**Table S2:** Follow‐up duration and measurement density by panel and diagnostic group.
**Table S3:** Reconciliation of baseline and longitudinal analytic samples.
**Table S4:** Characteristics of each biomarker‐specific analytic sample.
**Table S5:** Comparison of participants included in and excluded from each biomarker panel.
**Table S6:** Sensitivity of q‐values to the definition of the false discovery rate correction family.
**Table S7:** Longitudinal estimates under time‐varying age and under baseline age.
**Table S8:** Comparison of random‐intercept and random‐slope specifications.
**Table S9:** Residual diagnostics for baseline and longitudinal models.
**Table S10:** Variance inflation factors.
**Table S11:** Discriminative performance of each biomarker.
**Table S12:** Minimum detectable AD‐versus‐CU difference at 80% power.
**Table S13:** Baseline pairwise Spearman correlations with bootstrap confidence intervals.
**Table S14:** Diagnosis‐by‐APOE ε4 and diagnosis‐by‐sex interaction tests.
**Table S15:** Longitudinal models restricted to participants with two or more measurements.
**Table S16:** Within‐between decomposition of the time effect.
**Table S17:** Longitudinal models restricted to participants with at least 2 years of follow‐up.
**Table S18:** Diagnostic conversion during follow‐up.
**Table S19:** Longitudinal models with time‐varying diagnosis.
**Table S20:** Longitudinal models with the MCI group split into stable MCI and MCI‐to‐AD converters.
**Table S21:** Test for informative dropout.
**Table S22:** Assay quality flags in the vascular multiplex panel, by visit.
**Table S23:** Baseline vascular models with and without cardiometabolic and renal adjustment.
**Table S24:** Longitudinal vascular models with and without cardiometabolic and renal adjustment.
**Table S25:** Attenuation of the AD‐versus‐CU association with progressive covariate adjustment.
**Table S26:** Distribution of vascular risk factors by diagnostic group.

## Data Availability

The data used in this research were obtained from Alzheimer's Disease Neuroimaging Initiative (ADNI) and are available with permission to all researchers.
