# Supplementary material for: Glial and Vascular Plasma Biomarkers Across the Alzheimer's Disease Continuum: An ADNI‐Based Longitudinal Study
Source: FASEB Bioadv. 2026 Sep 19;8(9):e70155. doi: 10.1096/fba.2026-00194 (PMC13589249; doi:10.1096/fba.2026-00194)
Supplement: Supplementary file 1 [file FBA2-8-e70155-s001.docx]

**Table S1.** Number of biomarker measurements per participant, by panel and diagnostic group

| **Biomarker** | **Group** | **Measurements contributed** | **n** | **%** |
| --- | --- | --- | --- | --- |
| GFAP | CU | 1 measurement | 467 | 69.7 |
|  |  | 2 measurements | 179 | 26.7 |
|  |  | ≥3 measurements | 24 | 3.6 |
|  | MCI | 1 measurement | 296 | 68.8 |
|  |  | 2 measurements | 124 | 28.8 |
|  |  | ≥3 measurements | 10 | 2.3 |
|  | AD | 1 measurement | 105 | 69.1 |
|  |  | 2 measurements | 46 | 30.3 |
|  |  | ≥3 measurements | 1 | 0.7 |
| sTREM2 | CU | 1 measurement | 169 | 54.9 |
|  |  | 2 measurements | 56 | 18.2 |
|  |  | ≥3 measurements | 83 | 26.9 |
|  | MCI | 1 measurement | 276 | 52.3 |
|  |  | 2 measurements | 122 | 23.1 |
|  |  | ≥3 measurements | 130 | 24.6 |
|  | AD | 1 measurement | 163 | 83.6 |
|  |  | 2 measurements | 16 | 8.2 |
|  |  | ≥3 measurements | 16 | 8.2 |
| Vascular panel | CU | 1 measurement | 4 | 6.9 |
|  |  | 2 measurements | 54 | 93.1 |
|  | MCI | 1 measurement | 51 | 12.9 |
|  |  | 2 measurements | 345 | 87.1 |
|  | AD | 1 measurement | 16 | 14.3 |
|  |  | 2 measurements | 96 | 85.7 |

Counts and percentages of participants contributing one, two, or three or more measurements to each biomarker-specific longitudinal analytic sample. VEGF, sICAM-1 and sVCAM-1 share a single analytic sample and are shown once as the vascular panel. CU, cognitively unimpaired; MCI, mild cognitive impairment; AD, Alzheimer’s disease dementia.

**Table S2.** Follow-up duration and measurement density by panel and diagnostic group

| **Biomarker** | **Group** | **Participants** | **Mean visits** | **Median follow-up (y)** | **IQR (y)** | **Max (y)** | **≥2 visits (%)** |
| --- | --- | --- | --- | --- | --- | --- | --- |
| GFAP | CU | 670 | 1.34 | 0.00 | 0.00–2.05 | 19.25 | 30.3 |
|  | MCI | 430 | 1.33 | 0.00 | 0.00–1.87 | 10.14 | 31.2 |
|  | AD | 152 | 1.32 | 0.00 | 0.00–0.98 | 6.67 | 30.9 |
| sTREM2 | CU | 308 | 1.84 | 0.00 | 0.00–2.97 | 9.22 | 45.1 |
|  | MCI | 528 | 1.81 | 0.00 | 0.00–2.16 | 9.37 | 47.7 |
|  | AD | 195 | 1.28 | 0.00 | 0.00–0.00 | 4.16 | 16.4 |
| Vascular panel | CU | 58 | 1.93 | 1.02 | 0.99–1.06 | 1.34 | 93.1 |
|  | MCI | 396 | 1.87 | 1.00 | 0.97–1.03 | 1.63 | 87.1 |
|  | AD | 112 | 1.86 | 1.00 | 0.97–1.03 | 1.48 | 85.7 |

Median follow-up of 0.00 years indicates that more than half of participants in that group contributed a single measurement. Follow-up is the span between a participant’s first and last available measurement of that biomarker.

**Table S3.** Reconciliation of baseline and longitudinal analytic samples

| **Biomarker** | **Baseline n** | **Longitudinal n** | **Observations** | **With baseline value** | **Without** | **% without** | **Median first visit (y)** |
| --- | --- | --- | --- | --- | --- | --- | --- |
| GFAP | 832 | 1252 | 1671 | 831 | 421 | 33.6 | 4.07 |
| sTREM2 | 1001 | 1031 | 1771 | 1001 | 30 | 2.9 | 1.00 |
| Vascular panel | 566 | 566 | 1061 | 566 | 0 | 0.0 | - |

Longitudinal samples exceed baseline samples where participants without a biomarker measurement at the clinical baseline visit were included using later measurements. For those participants the diagnosis terms in the mixed-effects models are extrapolated to time zero. The final column gives the median time from baseline to the first available measurement among participants lacking a baseline value.

**Table S4.** Characteristics of each biomarker-specific analytic sample

| **Sample** | **Group** | **n** | **Age, mean (SD)** | **Female (%)** | **Education, mean (SD)** | **APOE ε4 (%)** |
| --- | --- | --- | --- | --- | --- | --- |
| GFAP (baseline model) | CU | 446 | 70.3 (7.4) | 61.4 | 16.7 (2.4) | 34.3 |
|  | MCI | 287 | 72.6 (7.4) | 44.3 | 16.0 (2.5) | 50.5 |
|  | AD | 99 | 76.0 (7.8) | 44.4 | 15.7 (2.5) | 67.7 |
| GFAP (longitudinal model) | CU | 673 | 72.6 (8.2) | 58.4 | 16.7 (2.4) | 35.2 |
|  | MCI | 380 | 74.0 (7.8) | 43.7 | 16.0 (2.6) | 48.2 |
|  | AD | 199 | 76.6 (8.4) | 40.7 | 15.9 (2.6) | 66.3 |
| sTREM2 (baseline model) | CU | 296 | 73.8 (6.0) | 50.7 | 16.4 (2.7) | 27.0 |
|  | MCI | 511 | 72.2 (7.5) | 40.7 | 16.0 (2.8) | 50.9 |
|  | AD | 194 | 74.7 (8.3) | 41.8 | 15.4 (2.8) | 67.0 |
| sTREM2 (longitudinal model) | CU | 308 | 73.9 (5.9) | 51.0 | 16.4 (2.7) | 27.3 |
|  | MCI | 525 | 72.3 (7.5) | 40.4 | 16.0 (2.8) | 51.2 |
|  | AD | 198 | 74.8 (8.3) | 41.9 | 15.4 (2.8) | 67.7 |
| sVCAM-1 (baseline model) | CU | 58 | 75.1 (5.8) | 48.3 | 15.7 (2.8) | 8.6 |
|  | MCI | 396 | 74.8 (7.5) | 35.4 | 15.6 (3.0) | 53.3 |
|  | AD | 112 | 74.9 (8.1) | 42.0 | 15.1 (3.2) | 67.9 |
| sVCAM-1 (longitudinal model) | CU | 58 | 75.1 (5.8) | 48.3 | 15.7 (2.8) | 8.6 |
|  | MCI | 396 | 74.8 (7.5) | 35.4 | 15.6 (3.0) | 53.3 |
|  | AD | 112 | 74.9 (8.1) | 42.0 | 15.1 (3.2) | 67.9 |
| full covariate-complete cohort | CU | 1206 | 71.2 (7.0) | 60.4 | 16.5 (2.6) | 32.0 |
|  | MCI | 1328 | 72.5 (7.7) | 43.4 | 15.9 (2.8) | 49.0 |
|  | AD | 472 | 74.7 (8.0) | 44.5 | 15.2 (2.9) | 67.0 |

Age and education are mean (SD). The vascular panel (VEGF, sICAM-1 and sVCAM-1) shares a single analytic sample and is shown once as sVCAM-1. Cognitively unimpaired participants constitute 53.6% of the GFAP baseline sample but only 10.2% of the vascular sample.

**Table S5.** Comparison of participants included in and excluded from each biomarker panel

| **Biomarker** | **Status** | **n** | **Age, mean (SD)** | **Female (%)** | **APOE ε4 (%)** | **CU / MCI / AD (%)** |
| --- | --- | --- | --- | --- | --- | --- |
| GFAP | included | 832 | 71.8 (7.7) | 53.5 | 43.9 | 53.6 / 34.5 / 11.9 |
|  | excluded | 2174 | 72.5 (7.5) | 49.3 | 46.1 | 35.0 / 47.9 / 17.2 |
| sTREM2 | included | 1001 | 73.2 (7.3) | 43.9 | 47.0 | 29.6 / 51.0 / 19.4 |
|  | excluded | 2005 | 71.9 (7.6) | 53.7 | 44.5 | 45.4 / 40.7 / 13.9 |
| Vascular panel | included | 566 | 74.8 (7.4) | 38.0 | 51.6 | 10.2 / 70.0 / 19.8 |
|  | excluded | 2440 | 71.7 (7.5) | 53.3 | 43.7 | 47.0 / 38.2 / 14.8 |

Panel A. Baseline characteristics. Diagnosis is shown as CU / MCI / AD percentages. Selection is strongly non-random and runs in opposite directions: the GFAP sample is enriched for cognitively unimpaired participants while the vascular sample is depleted of them.

**Table S5 (continued).** Comparison of participants included in and excluded from each biomarker panel

| **Biomarker** | **p, age** | **p, education** | **p, sex** | **p, APOE ε4** | **p, diagnosis** |
| --- | --- | --- | --- | --- | --- |
| GFAP | 0.013 | 1.66 × 10⁻⁵ | 0.042 | 0.305 | 1.18 × 10⁻¹⁹ |
| sTREM2 | 9.74 × 10⁻⁶ | 0.834 | 4.26 × 10⁻⁷ | 0.225 | 4.67 × 10⁻¹⁶ |
| Vascular panel | 1.56 × 10⁻¹⁸ | 4.03 × 10⁻⁵ | 6.71 × 10⁻¹¹ | 0.001 | 8.35 × 10⁻⁵⁹ |

Panel B. Tests of the included-versus-excluded contrast. Continuous variables were compared by t test and categorical variables by chi-square test.

**Table S6.** Sensitivity of q-values to the definition of the false discovery rate correction family

| **Model** | **Biomarker** | **Term** | **p** | **q, as published** | **q, reported terms** | **q, per biomarker** | **q, global** |
| --- | --- | --- | --- | --- | --- | --- | --- |
| Baseline | GFAP | AD vs CU | 1.66 × 10⁻¹⁵ | < 1 × 10⁻⁶ | < 1 × 10⁻⁶ | < 1 × 10⁻⁶ | < 1 × 10⁻⁶ |
|  | GFAP | MCI vs CU | 1.39 × 10⁻⁷ | < 1 × 10⁻⁶ | 1.00 × 10⁻⁶ | < 1 × 10⁻⁶ | 1.00 × 10⁻⁶ |
|  | sTREM2 | AD vs CU | 0.666 | 0.804 | 0.769 | 0.769 | 0.846 |
|  | sTREM2 | MCI vs CU | 0.769 | 0.842 | 0.769 | 0.769 | 0.851 |
|  | VEGF | AD vs CU | 0.005 | 0.012 | 0.013 | 0.010 | 0.013 |
|  | VEGF | MCI vs CU | 0.118 | 0.206 | 0.222 | 0.118 | 0.243 |
|  | sICAM-1 | AD vs CU | 0.228 | 0.332 | 0.325 | 0.455 | 0.347 |
|  | sICAM-1 | MCI vs CU | 0.705 | 0.822 | 0.769 | 0.705 | 0.851 |
|  | sVCAM-1 | AD vs CU | 5.93 × 10⁻⁴ | 0.001 | 0.002 | 0.001 | 0.002 |
|  | sVCAM-1 | MCI vs CU | 0.133 | 0.222 | 0.222 | 0.133 | 0.245 |
| Longitudinal | GFAP | AD vs CU at baseline | 2.54 × 10⁻²⁰ | < 1 × 10⁻⁶ | < 1 × 10⁻⁶ | < 1 × 10⁻⁶ | < 1 × 10⁻⁶ |
|  | GFAP | MCI vs CU at baseline | 1.87 × 10⁻⁸ | < 1 × 10⁻⁶ | < 1 × 10⁻⁶ | < 1 × 10⁻⁶ | < 1 × 10⁻⁶ |
|  | GFAP | Time (CU slope) | 2.12 × 10⁻⁴ | 5.59 × 10⁻⁴ | 6.64 × 10⁻⁴ | 3.54 × 10⁻⁴ | 7.43 × 10⁻⁴ |
|  | GFAP | Time × AD | 0.835 | 0.869 | 0.851 | 0.835 | 0.851 |
|  | GFAP | Time × MCI | 0.799 | 0.869 | 0.851 | 0.835 | 0.851 |
|  | sTREM2 | AD vs CU at baseline | 0.677 | 0.780 | 0.846 | 0.845 | 0.846 |
|  | sTREM2 | MCI vs CU at baseline | 0.521 | 0.668 | 0.724 | 0.845 | 0.730 |
|  | sTREM2 | Time (CU slope) | 0.195 | 0.335 | 0.324 | 0.486 | 0.324 |
|  | sTREM2 | Time × AD | 0.845 | 0.869 | 0.851 | 0.845 | 0.851 |
|  | sTREM2 | Time × MCI | 0.047 | 0.094 | 0.098 | 0.235 | 0.103 |
|  | VEGF | AD vs CU at baseline | 0.007 | 0.016 | 0.017 | 0.037 | 0.017 |
|  | VEGF | MCI vs CU at baseline | 0.133 | 0.255 | 0.255 | 0.332 | 0.245 |
|  | VEGF | Time (CU slope) | 0.740 | 0.822 | 0.851 | 0.851 | 0.851 |
|  | VEGF | Time × AD | 0.851 | 0.869 | 0.851 | 0.851 | 0.851 |
|  | VEGF | Time × MCI | 0.428 | 0.571 | 0.629 | 0.713 | 0.623 |
|  | sICAM-1 | AD vs CU at baseline | 0.207 | 0.345 | 0.324 | 0.259 | 0.330 |
|  | sICAM-1 | MCI vs CU at baseline | 0.654 | 0.778 | 0.846 | 0.654 | 0.846 |
|  | sICAM-1 | Time (CU slope) | 1.66 × 10⁻¹³ | < 1 × 10⁻⁶ | < 1 × 10⁻⁶ | < 1 × 10⁻⁶ | < 1 × 10⁻⁶ |
|  | sICAM-1 | Time × AD | 5.97 × 10⁻⁷ | 2.00 × 10⁻⁶ | 4.00 × 10⁻⁶ | 1.00 × 10⁻⁶ | 3.00 × 10⁻⁶ |
|  | sICAM-1 | Time × MCI | 1.11 × 10⁻⁵ | 3.10 × 10⁻⁵ | 4.00 × 10⁻⁵ | 1.90 × 10⁻⁵ | 4.30 × 10⁻⁵ |
|  | sVCAM-1 | AD vs CU at baseline | 7.61 × 10⁻⁴ | 0.002 | 0.002 | 9.52 × 10⁻⁴ | 0.002 |
|  | sVCAM-1 | MCI vs CU at baseline | 0.153 | 0.284 | 0.273 | 0.153 | 0.268 |
|  | sVCAM-1 | Time (CU slope) | 2.89 × 10⁻⁴ | 7.22 × 10⁻⁴ | 8.02 × 10⁻⁴ | 4.81 × 10⁻⁴ | 9.18 × 10⁻⁴ |
|  | sVCAM-1 | Time × AD | 6.75 × 10⁻⁶ | 2.00 × 10⁻⁵ | 2.80 × 10⁻⁵ | 1.70 × 10⁻⁵ | 3.00 × 10⁻⁵ |
|  | sVCAM-1 | Time × MCI | 2.17 × 10⁻⁶ | 7.00 × 10⁻⁶ | 1.10 × 10⁻⁵ | 1.10 × 10⁻⁵ | 1.10 × 10⁻⁵ |

Four family definitions are compared: as originally submitted (all coefficients of all five models within each model type, 35 baseline and 50 longitudinal tests, including intercepts); the reported terms only (10 baseline, 25 longitudinal); each biomarker separately; and globally across all 35 reported terms. No result changes significance status at q < 0.05 under any definition. q-values were stored to six decimal places, so values below 10⁻⁶ are shown as such.

**Table S7.** Longitudinal estimates under time-varying age and under baseline age

| **Biomarker** | **Term** | **% change, time-varying age** | **p** | **% change, baseline age** | **p** |
| --- | --- | --- | --- | --- | --- |
| GFAP | AD vs CU at baseline | 58.89 | 2.54 × 10⁻²⁰ | 58.89 | 2.54 × 10⁻²⁰ |
|  | MCI vs CU at baseline | 21.15 | 1.87 × 10⁻⁸ | 21.15 | 1.87 × 10⁻⁸ |
|  | Time (CU slope) | 1.48 | 2.12 × 10⁻⁴ | 4.50 | 4.04 × 10⁻³⁵ |
|  | Time × AD | 0.46 | 0.835 | 0.46 | 0.835 |
|  | Time × MCI | 0.15 | 0.799 | 0.15 | 0.799 |
| sTREM2 | AD vs CU at baseline | 2.07 | 0.677 | 2.07 | 0.677 |
|  | MCI vs CU at baseline | 2.45 | 0.521 | 2.45 | 0.521 |
|  | Time (CU slope) | 0.98 | 0.195 | 2.99 | 3.90 × 10⁻⁵ |
|  | Time × AD | 0.45 | 0.845 | 0.45 | 0.845 |
|  | Time × MCI | 1.81 | 0.047 | 1.81 | 0.047 |
| VEGF | AD vs CU at baseline | 13.62 | 0.008 | 13.62 | 0.008 |
|  | MCI vs CU at baseline | 6.40 | 0.135 | 6.40 | 0.135 |
|  | Time (CU slope) | -1.26 | 0.713 | -0.23 | 0.947 |
|  | Time × AD | 1.31 | 0.763 | 1.31 | 0.763 |
|  | Time × MCI | 3.09 | 0.411 | 3.09 | 0.411 |
| sICAM-1 | AD vs CU at baseline | 7.27 | 0.182 | 7.27 | 0.182 |
|  | MCI vs CU at baseline | -1.95 | 0.663 | -1.95 | 0.663 |
|  | Time (CU slope) | -23.62 | 1.79 × 10⁻¹² | -23.29 | 3.66 × 10⁻¹² |
|  | Time × AD | 24.65 | 3.09 × 10⁻⁶ | 24.65 | 3.09 × 10⁻⁶ |
|  | Time × MCI | 17.84 | 4.93 × 10⁻⁵ | 17.84 | 4.93 × 10⁻⁵ |
| sVCAM-1 | AD vs CU at baseline | 14.01 | 7.14 × 10⁻⁴ | 14.01 | 7.14 × 10⁻⁴ |
|  | MCI vs CU at baseline | 4.86 | 0.154 | 4.86 | 0.154 |
|  | Time (CU slope) | 7.66 | 3.41 × 10⁻⁴ | 8.64 | 5.80 × 10⁻⁵ |
|  | Time × AD | -11.02 | 6.69 × 10⁻⁶ | -11.02 | 6.69 × 10⁻⁶ |
|  | Time × MCI | -9.91 | 2.75 × 10⁻⁶ | -9.91 | 2.75 × 10⁻⁶ |

Age was originally constructed as baseline age plus time from baseline, making it perfectly collinear with the time variable within participants (within-person correlation 1.00 in all five models). The published time coefficient was therefore the rate of change net of the cross-sectional age effect. Two values change materially: the annual increase in cognitively unimpaired participants for GFAP and for sTREM2. All diagnosis-by-time interactions are numerically unchanged.

**Table S8.** Comparison of random-intercept and random-slope specifications

| **Biomarker** | **Participants, full** | **≥2 visits** | **≥3 visits** | **LRT χ²** | **p** | **Specification retained** |
| --- | --- | --- | --- | --- | --- | --- |
| GFAP | 1252 | 384 | 35 | 1.70 | 0.428 | random intercept retained |
| sTREM2 | 1031 | 423 | 229 | 5.06 | 0.080 | random intercept retained |
| VEGF | 566 | 495 | 0 | - | not estimable | random intercept retained |
| sICAM-1 | 566 | 495 | 0 | - | not estimable | random intercept retained |
| sVCAM-1 | 566 | 495 | 0 | - | not estimable | random intercept retained |

Because a majority of participants contribute a single measurement, a random slope cannot be estimated on the full sample. The likelihood ratio test was therefore performed among participants with two or more measurements. For the vascular panel, which comprises exactly two visits per participant, a random slope requires as many random effects as observations and is not identifiable.

**Table S9.** Residual diagnostics for baseline and longitudinal models

| **Biomarker** | **Baseline skewness** | **Baseline kurtosis** | **Non-constant variance p** | **Longitudinal skewness** | **Longitudinal kurtosis** |
| --- | --- | --- | --- | --- | --- |
| GFAP | -0.18 | 3.81 | 0.061 | -0.24 | 5.83 |
| sTREM2 | -0.22 | 3.08 | 0.610 | -0.08 | 3.95 |
| VEGF | 1.08 | 7.76 | 0.004 | 0.79 | 9.28 |
| sICAM-1 | -0.12 | 6.93 | 0.866 | 0.45 | 4.49 |
| sVCAM-1 | 0.38 | 3.49 | 0.169 | 0.21 | 5.45 |

Skewness near 0 and kurtosis near 3 indicate approximate normality. The non-constant variance test is Breusch-Pagan applied to the baseline model. VEGF baseline residuals are right-skewed with evidence of heteroscedasticity; heteroscedasticity-consistent standard errors did not alter the VEGF conclusions. Normal quantile plots are shown in Figure S2.

**Table S10.** Variance inflation factors

| **Biomarker** | **Model** | **Minimum VIF** | **Median VIF** | **Maximum VIF** |
| --- | --- | --- | --- | --- |
| GFAP | Baseline | 1.04 | 1.09 | 1.19 |
|  | Longitudinal | 1.04 | 1.09 | 2.46 |
| sTREM2 | Baseline | 1.07 | 1.10 | 1.14 |
|  | Longitudinal | 1.07 | 1.11 | 2.93 |
| VEGF | Baseline | 1.03 | 1.04 | 1.13 |
|  | Longitudinal | 1.03 | 1.13 | 11.42 |
| sICAM-1 | Baseline | 1.03 | 1.04 | 1.13 |
|  | Longitudinal | 1.03 | 1.13 | 11.37 |
| sVCAM-1 | Baseline | 1.03 | 1.04 | 1.13 |
|  | Longitudinal | 1.03 | 1.13 | 10.12 |

Minimum, median and maximum variance inflation factor across all predictors in each model. All baseline models are below 1.19. In the vascular longitudinal models the maximum reaches 9 to 11, which arises from the time and time-by-diagnosis product terms with an uncentred time variable; centring time reproduces identical interaction coefficients with all factors below 3.

**Table S11.** Discriminative performance of each biomarker

| **Biomarker** | **Contrast** | **n (case / control)** | **AUC (95% CI)** | **AUC, adjusted** | **Cohen’s d** | **Overlap** |
| --- | --- | --- | --- | --- | --- | --- |
| GFAP | AD vs CU | 99 / 446 | 0.822 (0.779–0.866) | 0.726 | 1.23 | 0.51 |
| GFAP | MCI vs CU | 287 / 446 | 0.631 (0.590–0.672) | 0.592 | 0.47 | 0.81 |
| sTREM2 | AD vs CU | 194 / 296 | 0.507 (0.455–0.559) | 0.500 | 0.08 | 0.92 |
| sTREM2 | MCI vs CU | 511 / 296 | 0.480 (0.438–0.521) | 0.496 | -0.04 | 0.93 |
| VEGF | AD vs CU | 112 / 58 | 0.612 (0.522–0.702) | 0.624 | 0.38 | 0.83 |
| VEGF | MCI vs CU | 396 / 58 | 0.549 (0.469–0.629) | 0.569 | 0.15 | 0.91 |
| sICAM-1 | AD vs CU | 112 / 58 | 0.528 (0.437–0.619) | 0.529 | 0.20 | 0.92 |
| sICAM-1 | MCI vs CU | 396 / 58 | 0.458 (0.379–0.537) | 0.463 | -0.08 | 0.90 |
| sVCAM-1 | AD vs CU | 112 / 58 | 0.625 (0.539–0.711) | 0.635 | 0.47 | 0.83 |
| sVCAM-1 | MCI vs CU | 396 / 58 | 0.529 (0.451–0.607) | 0.542 | 0.15 | 0.92 |

Area under the receiver operating characteristic curve with 95% confidence interval, and after residualisation on baseline age, sex, education and APOE ε4 status. The overlap coefficient is the proportion of the two distributions that coincide; a value of 1.00 indicates complete overlap. Only GFAP achieves discrimination of potential clinical interest.

**Table S12.** Minimum detectable AD-versus-CU difference at 80% power

| **Biomarker** | **n AD** | **n CU** | **Residual SD (log)** | **Minimum detectable (%)** | **Observed (%)** | **Powered** |
| --- | --- | --- | --- | --- | --- | --- |
| GFAP | 99 | 446 | 0.465 | 15.6 | 56.3 | Yes |
| sTREM2 | 194 | 296 | 0.518 | 14.3 | 2.2 | No |
| VEGF | 112 | 58 | 0.279 | 13.5 | 14.1 | Yes |
| sICAM-1 | 112 | 58 | 0.338 | 16.5 | 7.6 | No |
| sVCAM-1 | 112 | 58 | 0.233 | 11.1 | 14.7 | Yes |

Computed from the residual standard deviation of each adjusted baseline model and the two group sizes. The sICAM-1 and sTREM2 null results are uninformative about differences smaller than approximately 15% and should not be read as clean negatives.

**Table S13.** Baseline pairwise Spearman correlations with bootstrap confidence intervals

| **Biomarker 1** | **Biomarker 2** | **ρ** | **95% CI** | **Pairwise n** | **q** | **Precision** |
| --- | --- | --- | --- | --- | --- | --- |
| GFAP | sTREM2 | -0.01 | -0.21 to 0.20 | 94 | 0.941 | underpowered, exploratory |
|  | VEGF | -0.05 | -0.30 to 0.19 | 64 | 0.768 | underpowered, exploratory |
|  | sICAM-1 | 0.05 | -0.20 to 0.30 | 64 | 0.768 | underpowered, exploratory |
|  | sVCAM-1 | 0.21 | -0.04 to 0.45 | 64 | 0.199 | underpowered, exploratory |
| sTREM2 | VEGF | 0.08 | -0.04 to 0.19 | 266 | 0.357 | adequate |
|  | sICAM-1 | 0.07 | -0.06 to 0.19 | 266 | 0.407 | adequate |
|  | sVCAM-1 | 0.16 | 0.04 to 0.28 | 266 | 0.020 | adequate |
| VEGF | sICAM-1 | 0.31 | 0.23 to 0.38 | 566 | 9.34 × 10⁻¹³ | adequate |
|  | sVCAM-1 | 0.30 | 0.23 to 0.37 | 566 | 1.30 × 10⁻¹² | adequate |
| sICAM-1 | sVCAM-1 | 0.25 | 0.17 to 0.33 | 566 | 3.72 × 10⁻⁹ | adequate |

Confidence intervals from 2,000 bootstrap replicates. Benjamini-Hochberg correction was applied across the ten pairwise comparisons. Correlations based on fewer than 150 overlapping participants are flagged as underpowered; the small pairwise n reflects limited overlap between assay platforms rather than additional diagnostic exclusions. The GFAP-sVCAM-1 correlation is not statistically significant and its interval spans null to moderate values.

**Table S14.** Diagnosis-by-APOE ε4 and diagnosis-by-sex interaction tests

| **Biomarker** | **Interaction** | **LRT p** | **q** | **Interaction coefficients** |
| --- | --- | --- | --- | --- |
| GFAP | diagnosis x APOE ε4 | 0.198 | 0.917 | MCI:ε4: b=0.124, p=0.084; AD:ε4: b=-0.002, p=0.983 |
|  | diagnosis x sex | 0.953 | 0.953 | MCI:Male: b=-0.021, p=0.767; AD:Male: b=-0.018, p=0.865 |
| sTREM2 | diagnosis x APOE ε4 | 0.809 | 0.917 | MCI:ε4: b=0.039, p=0.636; AD:ε4: b=-0.013, p=0.903 |
|  | diagnosis x sex | 0.655 | 0.917 | MCI:Male: b=-0.049, p=0.516; AD:Male: b=0.024, p=0.805 |
| VEGF | diagnosis x APOE ε4 | 0.139 | 0.917 | MCI:ε4: b=-0.262, p=0.051; AD:ε4: b=-0.272, p=0.057 |
|  | diagnosis x sex | 0.429 | 0.917 | MCI:Male: b=-0.102, p=0.200; AD:Male: b=-0.101, p=0.266 |
| sICAM-1 | diagnosis x APOE ε4 | 0.819 | 0.917 | MCI:ε4: b=0.037, p=0.822; AD:ε4: b=-0.010, p=0.953 |
|  | diagnosis x sex | 0.648 | 0.917 | MCI:Male: b=0.053, p=0.584; AD:Male: b=0.100, p=0.364 |
| sVCAM-1 | diagnosis x APOE ε4 | 0.826 | 0.917 | MCI:ε4: b=-0.004, p=0.969; AD:ε4: b=-0.037, p=0.758 |
|  | diagnosis x sex | 0.607 | 0.917 | MCI:Male: b=0.035, p=0.594; AD:Male: b=-0.012, p=0.874 |

Likelihood ratio tests comparing the adjusted baseline model with and without the interaction. Benjamini-Hochberg correction was applied across the ten tests. No interaction is significant after correction.

**Table S15.** Longitudinal models restricted to participants with two or more measurements

| **Biomarker** | **Term** | **% change** | **p** | **Obs / participants** |
| --- | --- | --- | --- | --- |
| GFAP | Time (CU slope) | 5.74 | 2.82 × 10⁻²⁸ | 803 / 384 |
|  | MCI vs CU at baseline | 31.11 | 2.82 × 10⁻⁶ | 803 / 384 |
|  | AD vs CU at baseline | 70.14 | 1.16 × 10⁻¹⁰ | 803 / 384 |
|  | Time × MCI | -0.68 | 0.422 | 803 / 384 |
|  | Time × AD | -0.17 | 0.946 | 803 / 384 |
| sTREM2 | Time (CU slope) | 2.66 | 3.32 × 10⁻⁴ | 1163 / 423 |
|  | MCI vs CU at baseline | 1.24 | 0.820 | 1163 / 423 |
|  | AD vs CU at baseline | -5.50 | 0.578 | 1163 / 423 |
|  | Time × MCI | 1.83 | 0.049 | 1163 / 423 |
|  | Time × AD | 0.66 | 0.785 | 1163 / 423 |
| VEGF | Time (CU slope) | -0.48 | 0.889 | 990 / 495 |
|  | MCI vs CU at baseline | 4.50 | 0.307 | 990 / 495 |
|  | AD vs CU at baseline | 11.67 | 0.029 | 990 / 495 |
|  | Time × MCI | 3.89 | 0.306 | 990 / 495 |
|  | Time × AD | 2.16 | 0.623 | 990 / 495 |
| sICAM-1 | Time (CU slope) | -23.63 | 1.79 × 10⁻¹² | 990 / 495 |
|  | MCI vs CU at baseline | -3.95 | 0.384 | 990 / 495 |
|  | AD vs CU at baseline | 3.10 | 0.572 | 990 / 495 |
|  | Time × MCI | 18.82 | 2.11 × 10⁻⁵ | 990 / 495 |
|  | Time × AD | 26.80 | 5.75 × 10⁻⁷ | 990 / 495 |
| sVCAM-1 | Time (CU slope) | 8.62 | 6.46 × 10⁻⁵ | 990 / 495 |
|  | MCI vs CU at baseline | 4.98 | 0.162 | 990 / 495 |
|  | AD vs CU at baseline | 12.56 | 0.004 | 990 / 495 |
|  | Time × MCI | -9.90 | 3.18 × 10⁻⁶ | 990 / 495 |
|  | Time × AD | -10.70 | 1.43 × 10⁻⁵ | 990 / 495 |

Participants contributing a single observation contribute nothing to within-person slope estimation but do influence the pooled time coefficient. Restricting to participants with repeat measurements strengthens rather than weakens the GFAP time effect.

**Table S16.** Within-between decomposition of the time effect

| **Biomarker** | **Component** | **Term** | **% change** | **p** |
| --- | --- | --- | --- | --- |
| GFAP | within-person | Time, within-person | 6.08 | 2.35 × 10⁻²⁸ |
|  | between-person | Time, between-person | 3.52 | 4.55 × 10⁻²⁰ |
|  | within-person | Time, within-person:MCI | -0.56 | 0.548 |
|  | within-person | Time, within-person:AD | -0.31 | 0.906 |
| sTREM2 | within-person | Time, within-person | 2.59 | 5.12 × 10⁻⁴ |
|  | between-person | Time, between-person | 8.95 | 2.22 × 10⁻⁶ |
|  | within-person | Time, within-person:MCI | 2.00 | 0.033 |
|  | within-person | Time, within-person:AD | 0.63 | 0.795 |
| VEGF | within-person | Time, within-person | -0.67 | 0.847 |
|  | between-person | Time, between-person | -10.86 | 0.088 |
|  | within-person | Time, within-person:MCI | 4.07 | 0.285 |
|  | within-person | Time, within-person:AD | 2.30 | 0.601 |
| sICAM-1 | within-person | Time, within-person | -23.71 | 1.93 × 10⁻¹² |
|  | between-person | Time, between-person | -22.05 | 7.22 × 10⁻⁴ |
|  | within-person | Time, within-person:MCI | 18.85 | 2.29 × 10⁻⁵ |
|  | within-person | Time, within-person:AD | 27.20 | 4.75 × 10⁻⁷ |
| sVCAM-1 | within-person | Time, within-person | 8.60 | 6.64 × 10⁻⁵ |
|  | between-person | Time, between-person | -0.83 | 0.879 |
|  | within-person | Time, within-person:MCI | -9.97 | 2.67 × 10⁻⁶ |
|  | within-person | Time, within-person:AD | -10.74 | 1.33 × 10⁻⁵ |

The time variable was separated into a within-person component (deviation from each participant’s own mean) and a between-person component (the participant mean). The within-person component is the estimate of genuine longitudinal change. For GFAP it is 6.08% per year, larger than the 4.50% obtained from the pooled model.

**Table S17.** Longitudinal models restricted to participants with at least two years of follow-up

| **Biomarker** | **Term** | **% change** | **p** | **Obs / participants** |
| --- | --- | --- | --- | --- |
| GFAP | Time (CU slope) | 5.87 | 3.88 × 10⁻²⁶ | 622 / 294 |
|  | MCI vs CU at baseline | 26.34 | 3.74 × 10⁻⁴ | 622 / 294 |
|  | AD vs CU at baseline | 67.51 | 1.09 × 10⁻⁴ | 622 / 294 |
|  | Time × MCI | -0.42 | 0.655 | 622 / 294 |
|  | Time × AD | -0.18 | 0.950 | 622 / 294 |
| sTREM2 | Time (CU slope) | 2.36 | 0.001 | 951 / 323 |
|  | MCI vs CU at baseline | -2.45 | 0.672 | 951 / 323 |
|  | AD vs CU at baseline | -8.91 | 0.452 | 951 / 323 |
|  | Time × MCI | 1.76 | 0.058 | 951 / 323 |
|  | Time × AD | 3.15 | 0.253 | 951 / 323 |

This restriction is not possible for the vascular panel, whose maximum observed span is 1.63 years and in which no participant reaches two years of follow-up. That constraint is itself a limitation of the vascular longitudinal findings.

**Table S18.** Diagnostic conversion during follow-up

| **Baseline diagnosis** | **Conversion status** | **n** | **%** |
| --- | --- | --- | --- |
| CU | CU progressor | 178 | 11.3 |
| CU | stable CU | 1401 | 88.7 |
| MCI | MCI to AD converter | 404 | 25.0 |
| MCI | stable MCI | 1211 | 75.0 |
| AD | AD at baseline | 568 | 100.0 |

Conversion was determined from visit-level diagnosis. Within the biomarker panels, the proportion of baseline MCI participants converting to AD was 29.8% for GFAP, 41.3% for sTREM2 and 54.0% for the vascular panel.

**Table S19.** Longitudinal models with time-varying diagnosis

| **Biomarker** | **Term** | **% change** | **p** | **Obs / participants** |
| --- | --- | --- | --- | --- |
| GFAP | Time (CU slope) | 4.18 | 1.12 × 10⁻²⁴ | 1671 / 1252 |
|  | MCI vs CU | 20.35 | 1.49 × 10⁻⁸ | 1671 / 1252 |
|  | AD vs CU | 54.62 | 2.36 × 10⁻²² | 1671 / 1252 |
|  | years_from_baseline:MCI | -0.60 | 0.353 | 1671 / 1252 |
|  | years_from_baseline:AD | -1.53 | 0.069 | 1671 / 1252 |
| sTREM2 | Time (CU slope) | 3.42 | 5.72 × 10⁻⁶ | 1771 / 1031 |
|  | MCI vs CU | -2.44 | 0.429 | 1771 / 1031 |
|  | AD vs CU | 4.59 | 0.277 | 1771 / 1031 |
|  | years_from_baseline:MCI | 1.50 | 0.133 | 1771 / 1031 |
|  | years_from_baseline:AD | -0.91 | 0.477 | 1771 / 1031 |
| VEGF | Time (CU slope) | 0.32 | 0.926 | 1061 / 566 |
|  | MCI vs CU | 5.85 | 0.157 | 1061 / 566 |
|  | AD vs CU | 12.28 | 0.012 | 1061 / 566 |
|  | years_from_baseline:MCI | 2.05 | 0.583 | 1061 / 566 |
|  | years_from_baseline:AD | 0.16 | 0.970 | 1061 / 566 |
| sICAM-1 | Time (CU slope) | -22.46 | 1.35 × 10⁻¹¹ | 1061 / 566 |
|  | MCI vs CU | -3.06 | 0.481 | 1061 / 566 |
|  | AD vs CU | 1.15 | 0.820 | 1061 / 566 |
|  | years_from_baseline:MCI | 16.05 | 2.47 × 10⁻⁴ | 1061 / 566 |
|  | years_from_baseline:AD | 19.73 | 8.03 × 10⁻⁵ | 1061 / 566 |
| sVCAM-1 | Time (CU slope) | 8.10 | 1.28 × 10⁻⁴ | 1061 / 566 |
|  | MCI vs CU | 7.67 | 0.016 | 1061 / 566 |
|  | AD vs CU | 11.81 | 0.001 | 1061 / 566 |
|  | years_from_baseline:MCI | -9.32 | 1.25 × 10⁻⁵ | 1061 / 566 |
|  | years_from_baseline:AD | -11.63 | 1.08 × 10⁻⁶ | 1061 / 566 |

Diagnosis was modelled at each visit rather than as a fixed baseline label. All findings are reproduced.

**Table S20.** Longitudinal models with the MCI group split into stable MCI and MCI-to-AD converters

| **Biomarker** | **Interaction term** | **% change per year** | **p** |
| --- | --- | --- | --- |
| GFAP | Time × stable MCI | -0.54 | 0.426 |
|  | Time × MCI converter | 0.69 | 0.441 |
|  | Time × AD | 0.41 | 0.852 |
| sTREM2 | Time × stable MCI | 2.04 | 0.060 |
|  | Time × MCI converter | 1.53 | 0.145 |
|  | Time × AD | 0.46 | 0.841 |
| VEGF | Time × stable MCI | 3.76 | 0.359 |
|  | Time × MCI converter | 2.54 | 0.519 |
|  | Time × AD | 1.31 | 0.763 |
| sICAM-1 | Time × stable MCI | 20.24 | 2.80 × 10⁻⁵ |
|  | Time × MCI converter | 16.11 | 4.19 × 10⁻⁴ |
|  | Time × AD | 24.65 | 3.01 × 10⁻⁶ |
| sVCAM-1 | Time × stable MCI | -9.75 | 2.28 × 10⁻⁵ |
|  | Time × MCI converter | -10.00 | 6.40 × 10⁻⁶ |
|  | Time × AD | -11.02 | 6.70 × 10⁻⁶ |

Only the diagnosis-by-time interaction terms are shown. Stable MCI and converting MCI behave almost identically for both adhesion molecules, so conversion heterogeneity does not generate the observed attenuation pattern.

**Table S21.** Test for informative dropout

| **Biomarker** | **Predictor** | **Odds ratio** | **p** |
| --- | --- | --- | --- |
| GFAP | baseline biomarker level (log) | 1.02 | 0.871 |
|  | MCI vs CU | 0.94 | 0.662 |
|  | AD vs CU | 0.86 | 0.472 |
| sTREM2 | baseline biomarker level (log) | 1.32 | 0.028 |
|  | MCI vs CU | 1.18 | 0.269 |
|  | AD vs CU | 0.25 | 3.47 × 10⁻⁹ |
| VEGF | baseline biomarker level (log) | 0.34 | 0.013 |
|  | MCI vs CU | 0.54 | 0.264 |
|  | AD vs CU | 0.51 | 0.277 |
| sICAM-1 | baseline biomarker level (log) | 0.45 | 0.036 |
|  | MCI vs CU | 0.50 | 0.211 |
|  | AD vs CU | 0.47 | 0.217 |
| sVCAM-1 | baseline biomarker level (log) | 0.67 | 0.462 |
|  | MCI vs CU | 0.51 | 0.232 |
|  | AD vs CU | 0.47 | 0.223 |

Logistic models of whether a participant returned for a second measurement, adjusted for baseline diagnosis, baseline age, sex, education and APOE ε4 status. An odds ratio below 1 indicates lower probability of return. Baseline GFAP does not predict return. AD participants were substantially less likely to return in the sTREM2 panel, and higher baseline sICAM-1 and VEGF predicted non-return.

**Table S22.** Assay quality flags in the vascular multiplex panel, by visit

| **Biomarker** | **Visit** | **n samples** | **Below detection limit (%)** | **Low read (%)** | **Outlier (%)** |
| --- | --- | --- | --- | --- | --- |
| VEGF | Baseline | 566 | 0.00 | 0.00 | 0.20 |
|  | Month 12 | 497 | 0.00 | 0.00 | 0.40 |
| sICAM-1 | Baseline | 566 | 0.00 | 0.00 | 0.20 |
|  | Month 12 | 497 | 0.00 | 0.00 | 0.20 |
| sVCAM-1 | Baseline | 566 | 0.00 | 0.00 | 0.00 |
|  | Month 12 | 497 | 0.00 | 0.00 | 0.00 |

Flag rates from the raw multiplex release. Rates are negligible and balanced between the two visits, and excluding flagged samples left all model estimates unchanged (sICAM-1 Time × AD moved from 24.65% to 24.46%). The release carries no plate or assay-run identifier, so a run-to-run analytical effect can be neither confirmed nor excluded.

**Table S23.** Baseline vascular models with and without cardiometabolic and renal adjustment

| **Biomarker** | **Tier** | **Contrast** | **% difference (95% CI)** | **p** | **n** |
| --- | --- | --- | --- | --- | --- |
| VEGF | 1: as published | AD vs CU | 14.13 (4.0 to 25.3) | 0.006 | 566 |
|  |  | MCI vs CU | 6.59 (-1.6 to 15.5) | 0.119 | 566 |
|  | 2: + blood pressure and meds | AD vs CU | 17.38 (7.1 to 28.6) | 6.47 × 10⁻⁴ | 566 |
|  |  | MCI vs CU | 8.16 (0.0 to 17.0) | 0.050 | 566 |
|  | 3: + BMI and smoking | AD vs CU | 16.84 (6.5 to 28.2) | 0.001 | 562 |
|  |  | MCI vs CU | 7.67 (-0.6 to 16.6) | 0.070 | 562 |
|  | 4: + renal function | AD vs CU | 12.37 (2.6 to 23.1) | 0.012 | 550 |
|  |  | MCI vs CU | 5.96 (-2.0 to 14.5) | 0.145 | 550 |
| sICAM-1 | 1: as published | AD vs CU | 7.59 (-3.9 to 20.4) | 0.204 | 566 |
|  |  | MCI vs CU | -1.67 (-10.8 to 8.3) | 0.733 | 566 |
|  | 2: + blood pressure and meds | AD vs CU | 7.72 (-3.9 to 20.7) | 0.201 | 566 |
|  |  | MCI vs CU | -1.57 (-10.7 to 8.5) | 0.751 | 566 |
|  | 3: + BMI and smoking | AD vs CU | 7.75 (-4.0 to 20.9) | 0.206 | 562 |
|  |  | MCI vs CU | -1.87 (-11.2 to 8.4) | 0.710 | 562 |
|  | 4: + renal function | AD vs CU | 4.54 (-7.0 to 17.6) | 0.459 | 550 |
|  |  | MCI vs CU | -2.57 (-11.9 to 7.7) | 0.610 | 550 |
| sVCAM-1 | 1: as published | AD vs CU | 14.70 (6.1 to 24.0) | 5.75 × 10⁻⁴ | 566 |
|  |  | MCI vs CU | 5.24 (-1.6 to 12.5) | 0.134 | 566 |
|  | 2: + blood pressure and meds | AD vs CU | 15.93 (7.2 to 25.3) | 2.26 × 10⁻⁴ | 566 |
|  |  | MCI vs CU | 5.86 (-1.0 to 13.2) | 0.096 | 566 |
|  | 3: + BMI and smoking | AD vs CU | 15.86 (7.0 to 25.4) | 2.90 × 10⁻⁴ | 562 |
|  |  | MCI vs CU | 5.75 (-1.2 to 13.2) | 0.108 | 562 |
|  | 4: + renal function | AD vs CU | 12.28 (3.8 to 21.4) | 0.004 | 550 |
|  |  | MCI vs CU | 4.58 (-2.2 to 11.8) | 0.189 | 550 |

Tier 1 uses the covariates of the primary analysis (baseline age, sex, education, APOE ε4). Tier 2 adds systolic blood pressure and antihypertensive and antidiabetic medication use; tier 3 adds body mass index and smoking history; tier 4 adds estimated glomerular filtration rate. Each tier is fitted on its own complete-case sample. The AD-versus-CU associations for VEGF and sVCAM-1 survive full adjustment.

**Table S24.** Longitudinal vascular models with and without cardiometabolic and renal adjustment

| **Biomarker** | **Tier** | **Term** | **% change per year** | **p** | **Obs / participants** |
| --- | --- | --- | --- | --- | --- |
| VEGF | 1: as published | Time (CU slope) | -0.23 | 0.947 | 1061 / 566 |
|  |  | Time × MCI | 3.09 | 0.411 | 1061 / 566 |
|  |  | Time × AD | 1.31 | 0.763 | 1061 / 566 |
|  | 2: + blood pressure and meds | Time (CU slope) | -0.50 | 0.883 | 1061 / 566 |
|  |  | Time × MCI | 3.25 | 0.388 | 1061 / 566 |
|  |  | Time × AD | 1.61 | 0.711 | 1061 / 566 |
|  | 3: + BMI and smoking | Time (CU slope) | -0.95 | 0.783 | 1055 / 562 |
|  |  | Time × MCI | 3.75 | 0.325 | 1055 / 562 |
|  |  | Time × AD | 2.04 | 0.642 | 1055 / 562 |
|  | 4: + renal function | Time (CU slope) | -1.03 | 0.768 | 1034 / 550 |
|  |  | Time × MCI | 3.80 | 0.326 | 1034 / 550 |
|  |  | Time × AD | 2.29 | 0.608 | 1034 / 550 |
| sICAM-1 | 1: as published | Time (CU slope) | -23.29 | 3.66 × 10⁻¹² | 1061 / 566 |
|  |  | Time × MCI | 17.84 | 4.93 × 10⁻⁵ | 1061 / 566 |
|  |  | Time × AD | 24.65 | 3.09 × 10⁻⁶ | 1061 / 566 |
|  | 2: + blood pressure and meds | Time (CU slope) | -23.30 | 3.65 × 10⁻¹² | 1061 / 566 |
|  |  | Time × MCI | 17.83 | 4.94 × 10⁻⁵ | 1061 / 566 |
|  |  | Time × AD | 24.65 | 3.07 × 10⁻⁶ | 1061 / 566 |
|  | 3: + BMI and smoking | Time (CU slope) | -23.07 | 1.00 × 10⁻¹¹ | 1055 / 562 |
|  |  | Time × MCI | 17.60 | 7.05 × 10⁻⁵ | 1055 / 562 |
|  |  | Time × AD | 24.27 | 4.84 × 10⁻⁶ | 1055 / 562 |
|  | 4: + renal function | Time (CU slope) | -23.20 | 1.42 × 10⁻¹¹ | 1034 / 550 |
|  |  | Time × MCI | 17.60 | 8.89 × 10⁻⁵ | 1034 / 550 |
|  |  | Time × AD | 24.76 | 4.54 × 10⁻⁶ | 1034 / 550 |
| sVCAM-1 | 1: as published | Time (CU slope) | 8.64 | 5.80 × 10⁻⁵ | 1061 / 566 |
|  |  | Time × MCI | -9.91 | 2.75 × 10⁻⁶ | 1061 / 566 |
|  |  | Time × AD | -11.02 | 6.69 × 10⁻⁶ | 1061 / 566 |
|  | 2: + blood pressure and meds | Time (CU slope) | 8.60 | 6.17 × 10⁻⁵ | 1061 / 566 |
|  |  | Time × MCI | -9.90 | 2.84 × 10⁻⁶ | 1061 / 566 |
|  |  | Time × AD | -10.98 | 7.24 × 10⁻⁶ | 1061 / 566 |
|  | 3: + BMI and smoking | Time (CU slope) | 8.59 | 6.98 × 10⁻⁵ | 1055 / 562 |
|  |  | Time × MCI | -9.96 | 2.71 × 10⁻⁶ | 1055 / 562 |
|  |  | Time × AD | -10.97 | 7.81 × 10⁻⁶ | 1055 / 562 |
|  | 4: + renal function | Time (CU slope) | 8.66 | 6.66 × 10⁻⁵ | 1034 / 550 |
|  |  | Time × MCI | -9.74 | 5.02 × 10⁻⁶ | 1034 / 550 |
|  |  | Time × AD | -11.02 | 7.96 × 10⁻⁶ | 1034 / 550 |

Tiers as defined in Table S23. Only the time and diagnosis-by-time terms are shown. The diagnosis-by-time interactions for sICAM-1 and sVCAM-1 are essentially unchanged across all four tiers, and all remain significant at p < 10⁻⁴ on 550 participants after full adjustment.

**Table S25.** Attenuation of the AD-versus-CU association with progressive covariate adjustment

| **Biomarker** | **Tier** | **β, AD vs CU** | **n** | **Attenuation vs tier 1 (%)** |
| --- | --- | --- | --- | --- |
| VEGF | 1: as published | 0.1321 | 566 | 0.0 |
| sICAM-1 | 1: as published | 0.0731 | 566 | 0.0 |
| sVCAM-1 | 1: as published | 0.1372 | 566 | 0.0 |
| VEGF | 2: + blood pressure and meds | 0.1602 | 566 | -21.2 |
| sICAM-1 | 2: + blood pressure and meds | 0.0744 | 566 | -1.7 |
| sVCAM-1 | 2: + blood pressure and meds | 0.1479 | 566 | -7.8 |
| VEGF | 3: + BMI and smoking | 0.1557 | 562 | -17.8 |
| sICAM-1 | 3: + BMI and smoking | 0.0746 | 562 | -2.0 |
| sVCAM-1 | 3: + BMI and smoking | 0.1472 | 562 | -7.3 |
| VEGF | 4: + renal function | 0.1166 | 550 | 11.7 |
| sICAM-1 | 4: + renal function | 0.0444 | 550 | 39.3 |
| sVCAM-1 | 4: + renal function | 0.1158 | 550 | 15.6 |

Negative attenuation indicates that the estimate increased with adjustment. Of the three markers, sICAM-1 is the most sensitive to renal adjustment, although it was not significantly associated with diagnosis in any specification.

**Table S26.** Distribution of vascular risk factors by diagnostic group

| **Group** | **n** | **Systolic BP (mmHg)** | **Antihypertensive (%)** | **Antidiabetic (%)** | **BMI** | **Smoking (%)** | **eGFR** |
| --- | --- | --- | --- | --- | --- | --- | --- |
| CU | 1206 | 134.4 (n=1205) | 55.0 (n=1192) | 13.0 | 29.6 (n=1201) | 40.0 | 73.5 (n=433) |
| MCI | 1328 | 134.7 (n=1328) | 54.0 (n=1311) | 12.0 | 29.1 (n=1324) | 39.0 | 74.0 (n=854) |
| AD | 472 | 136.0 (n=472) | 47.0 (n=468) | 10.0 | 28.9 (n=470) | 39.0 | 70.8 (n=311) |

Means with the number of participants contributing each variable. Medication use and smoking are percentages. The risk factors are closely balanced across diagnostic groups, which accounts for the small movement in estimates on adjustment.
