# Supplementary material for: Glial and Vascular Plasma Biomarkers Across the Alzheimer's Disease Continuum: An ADNI‐Based Longitudinal Study
Source: FASEB Bioadv. 2026 Sep 19;8(9):e70155. doi: 10.1096/fba.2026-00194 (PMC13589249; doi:10.1096/fba.2026-00194)
Supplement: Supplementary file 2 [file FBA2-8-e70155-s002.docx]

**Figure S1.** Study flow and biomarker-specific analytic cohorts. Flow diagram showing construction of the ADNI clinical core cohort and the biomarker-specific analytic samples. Participants were excluded for missing baseline visit, missing baseline CU/MCI/AD diagnosis, or incomplete required baseline covariates. Baseline and longitudinal model sample sizes are shown separately for each biomarker. CU, cognitively unimpaired; MCI, mild cognitive impairment; AD, Alzheimer's disease dementia; APOE4, apolipoprotein E ε4 carrier status.

**Figure S2.** Normal quantile plots of baseline model residuals. Residuals from the covariate-adjusted baseline linear regression model for each biomarker, plotted against theoretical normal quantiles. Points falling along the reference line indicate approximate normality. VEGF residuals depart from the line in the upper tail, consistent with the right skew and non-constant variance reported in Table S9.

**Figure S3.** Within-person plasma GFAP trajectories. Individual trajectories for participants contributing two or more GFAP measurements (n = 384). Grey lines represent individual participants; coloured lines and shaded ribbons show fitted group trends with 95% confidence intervals by baseline diagnosis. The within-person increase is 6.08% per year, larger than the 4.50% obtained from the pooled model that also includes single-measurement participants.
